# Discordance Between the Predicted Versus the Actually Recognized CD8+ T Cell Epitopes of HCMV pp65 Antigen and Aleatory Epitope Dominance

**DOI:** 10.3389/fimmu.2020.618428

**Published:** 2021-02-09

**Authors:** Alexander A. Lehmann, Ting Zhang, Pedro A. Reche, Paul V. Lehmann

**Affiliations:** ^1^ Research and Development, Cellular Technology Ltd., Shaker Heights, OH, United States; ^2^ Laboratorio de Inmunomedicina & Inmunoinformatica, Departamento de Immunologia & O2, Facultad de Medicina, Universidad Complutense de Madrid, Madrid, Spain

**Keywords:** epitope prediction, brute force epitope mapping, high throughput, ImmunoSpot, ELISPOT

## Abstract

CD8+ T cell immune monitoring aims at measuring the size and functions of antigen-specific CD8+ T cell populations, thereby providing insights into cell-mediated immunity operational in a test subject. The selection of peptides for *ex vivo* CD8+ T cell detection is critical because within a complex antigen exists a multitude of potential epitopes that can be presented by HLA class I molecules. Further complicating this task, there is HLA class I polygenism and polymorphism which predisposes CD8+ T cell responses towards individualized epitope recognition profiles. In this study, we compare the actual CD8+ T cell recognition of a well-characterized model antigen, human cytomegalovirus (HCMV) pp65 protein, with its anticipated epitope coverage. Due to the abundance of experimentally defined HLA-A^*^02:01-restricted pp65 epitopes, and because *in silico* epitope predictions are most advanced for HLA-A^*^02:01, we elected to focus on subjects expressing this allele. In each test subject, every possible CD8+ T cell epitope was systematically covered testing 553 individual peptides that walk the sequence of pp65 in steps of single amino acids. Highly individualized CD8+ T cell response profiles with aleatory epitope recognition patterns were observed. No correlation was found between epitopes’ ranking on the prediction scale and their actual immune dominance. Collectively, these data suggest that accurate CD8+ T cell immune monitoring may necessitate reliance on agnostic mega peptide pools, or brute force mapping, rather than electing individual peptides as representative epitopes for tetramer and other multimer labeling of surface antigen receptors.

## Introduction

T cell immune monitoring has a long and successful track record in murine models in which defined experimental conditions, small model antigens, and work with inbred mouse strains expressing few restriction elements (MHC molecules) simplified the task (1). The magnitude of scope is entirely different when the outbred human population is to be studied, largely due to the immense diversity in complexity restriction elements (human leukocyte antigens, HLA) and the of the antigenic systems, such as viruses. To comprehensively monitor T cell immunity to SARS-CoV-2, for example, this virus’ entire proteome, 9,871 amino acids long (2), would need to be considered. In this report, we confined ourselves to a single protein of HCMV, pp65, which is “only” 561 amino acid long, and to subjects who shared a common HLA-A^*^02:01 restriction element, but differed in the remaining HLA class I alleles.

Monitoring CD4+ T cell immunity is relatively simple. When the test antigen of interest is added as a protein to peripheral blood mononuclear cells (PBMC), the antigen presenting cells (APC) contained in the PBMC will acquire, process, and present the antigen (3). Unfortunately, this is not the case for *ex vivo* CD8+ T cell detection. CD8+ T cells evolved to recognize antigens actively bio-synthetized within host cells, as opposed to antigens that APC acquire from their surroundings. Thereby CD8+ T cells can survey ongoing protein synthesis in the cells of the body, permitting them to identify virally-infected or malignant cells, so as to kill them. During protein synthesis, defective byproducts also arise that are degraded by the proteasome into peptide fragments. Such peptides are loaded onto HLA class I molecules, and transported to the cell surface to be displayed to CD8+ T cells (4). Protein antigens are not suited to recall *in vivo*-primed CD8+ T cells within PBMC because exogenously added proteins are not efficiently presented to CD8+ T cells in the context of class I molecules. Instead, the CD8+ T cell epitopes need to be added as 8–11 amino acid long peptides that they can bind directly to cell surface expressed HLA class I molecules. From this requirement the need arises to select the “right” peptides for *ex vivo* CD8+ T cell immune monitoring: those very same epitopes that have induced a CD8+ T cell response *in vivo.* Missing the “right” peptides, or only partially covering them, has the consequence that the antigen-specific CD8+ T cell repertoire could go partially or entirely undetected.

Selecting the “right” peptides for CD8+ T cell immune monitoring is an inherently intricate task. HLA class I molecules are encoded by three genetic loci, HLA-A, HLA-B, and HLA-C, for which a multitude of alleles exist in the human population (5). Each allelic HLA class I molecule has a unique peptide binding specificity (6). As there are barely two humans with an identical HLA-type, there should be barely two humans who present the same array of epitopes. Protecting the species, T cell epitope recognition evolved to be highly individualized (7). Peptide selection for comprehensive CD8+ T cell immune monitoring must therefore account for the unique HLA allele composition in each test subject.

A mainstream effort for identifying the “right” peptides for CD8+ T cell monitoring is reliant upon *in silico* epitope predictions. As the peptide binding motifs for most HLA alleles are well-defined, predictions can be made as far as which peptide sequence of an antigen can bind to a given HLA allele, thus constituting a potential T cell epitope. Search engines have been made available to the scientific community to rank peptide sequences for their predicted binding strength to most HLA alleles, thus narrowing in on a finite set of epitopes. A critical assumption for epitope predictions is that peptides that rank high in their respective HLA allele binding score will be those that are being targeted most by CD8+ T cells. The data presented in this study challenge this hypothesis supporting the conclusions reached by Mei et al. (8).

Beyond doubt, a peptide needs to be able to bind to an HLA allele to be a candidate for T cell recognition. However, whether a peptide sequence of a protein antigen that has HLA-binding potential indeed becomes an epitope recognized by T cells is defined by many additional factors (9). Limitations exist on the level of antigen presentation, including whether that exact peptide is indeed generated through natural antigen processing, and whether it is produced in quantities that can outcompete other peptides, including self-peptides, for binding to the respective HLA molecules. It has been shown that different class I alleles present in an individual can compete with each other for epitope dominance (10). Limitations also exist on the level of the pre-immune T cell repertoire available to engage in antigen recognition. The duration and abundance of epitope presentation will also affect the ensuing CD8+ T cell response, being regulated both by a virus’ replication biology, and the host’s ability to control the virus. The CD8+ T cell response is dynamic (11). Therefore, it can be expected that only a fraction of peptides with HLA class I binding properties will elicit strong CD8+ T cell responses, becoming dominant epitopes. Other presented peptides might induce a weaker, subdominant, barely detectable, cryptic, or no CD8+ T cell responses at all. As all antigen-specific CD8+ T cells can be expected to contribute equally to the host’s defense, irrespective of their fine specificity, comprehensive immune monitoring must not focus on a single or few epitopes, but should instead accommodate all epitopes of an antigen targeted by CD8+ T cells in an individual in order to assess the entire antigen-specific T cell pool.

Next to predictions *in silico*, experimentally verified epitopes have been used as a guide to select peptides for CD8+ T cell immune monitoring. Over the years, T cell lines and clones specific for many viral antigens have been isolated and their epitope specificity compiled in databases (12, 13). Selection of such previously verified epitopes for immune monitoring is based on the assumption that epitope recognition, including epitope hierarchy, is constant in subjects who express the corresponding HLA allele. In other words, if an HLA-X-restricted peptide Y has been identified as an immune dominant epitope in an HLA-X positive subject Z, this peptide Y will also be immune dominant in HLA-X positive subjects V and W. Such predictable immune dominance prevails in simple murine models when inbred mice are studied that express minimal restriction element diversity (14). However, predictable epitope dominance is lost as soon as restriction element diversity rises through interbreeding these inbred mouse strains. In such F1 mice, aleatory epitope recognition prevails (15): T cells in each F1 mouse respond in an unpredictable, dice-like fashion (*alea* means dice in Latin) to epitopes to which the parental strains responded predictably. Aleatory epitope dominance may also apply to humans due to their diverse restriction element makeup (16). Therefore, in the present study of HCMV pp65 epitope recognition in HLA-A^*^02:01-positive individuals, we also compare the peptides that the CD8+ T cells actually target in our cohort with previously verified epitopes.

The third approach for CD8+ T cell immune monitoring is not to select peptides at all, but to systematically test all possible peptides of the antigen. This can be done by using mega peptide pools consisting of hundreds of peptides that cover entire proteins of a virus. By necessity, this approach has become standard recently in the first real world challenge on clinical T cell immune monitoring: trying to study T cell immunity induced by SARS-CoV-2 infection. This crude approach is simple and practical, yet permits comprehensive assessment of the entire expressed antigen-specific T cell repertoire in outbred populations, without requiring customization to HLA types of individuals, but it does not reveal the epitope specificity of the antigen-reactive T cells.

In this study, we applied an agnostic approach in which all possible peptides were tested individually on each subject in a “brute force” high-throughput manner (17). The ability to test hundreds, even thousands of peptides individually on a subject is a recent technological advancement. The hurdles that needed to be overcome included limitations in PBMC numbers available from a subject, access to extensive custom peptide libraries, high-throughput-capable T cell assay platforms, and automated data analysis. We have developed and report here large-scale epitope mapping strategies that can be readily adopted even in small academic laboratories operating on tight budgets. Empowered by the ability to experimentally verify CD8+ T cell epitope utilization at the highest possible resolution in the well-studied HCMV pp65 T cell immune monitoring model, we set out to compare the epitopes actually recognized with those that are predicted, or assumed to be recognized based on existing data.

A protein of HCMV, pp65 is as far as CD8+ T cell recognition goes arguably one of the best studied model antigens: over the decades, 31 epitopes have been experimentally verified for the HLA-A2 allele alone (these are listed in **Supplementary Table 1**, including the corresponding references). Moreover, while the understanding of the rules for peptide binding to most HLA alleles is overall advanced, they are by far best established for HLA-A2. In this study, therefore we focus on the actual CD8+ T cell recognition of pp65 epitopes in HLA-A2 positive subjects, asking the question whether previously defined epitopes or binding predictions suffice to guide the selection of individual peptides for immune monitoring purposes. We assume that lessons learned from the best studied model antigen have implications for still less characterized foreign antigens, such as those of the SARS-CoV-2 virus, or tumor/self-antigens. We draw attention to how incomplete our appreciation of an individual’s expressed epitope space currently is even when it comes to a well-studied foreign antigen. Epitope utilization in anti-self/cancer antigen recognition can be expected to underly the same rules, plus T cell repertoire limitations caused by negative selection by abundantly presented self-peptides. Our data suggest that neither epitope predictions, nor reliance on known epitopes suffice, but rather that the agnostic route is best suited for comprehensive CD8+ T cell immune monitoring for foreign antigens, and by inference, tumor antigens as well.

HLA multimers (tetramers, pentamers, dextramers) are frequently used for CD8+ T cell monitoring. Consisting of HLA molecules loaded with a peptide epitope, multimers constitute the T cell-receptor (TCR) ligand that can be used to selectively stain antigen-specific T cells (18). This technique is invaluable for the in-depth phenotypic analysis of the antigen-specific T cells *via* flow cytometry (19), but its limitation is that it requires epitope utilization to be predictable, either based on previously defined, or on in silico predicted epitopes. The data communicated in the following call into question whether such predictions are accurate even in the case of the arguably best-defined model antigen, pp65. We argue that the agnostic use of peptide libraries is needed for reliable CD8+ T cell monitoring, along with the utilization of techniques that are suited for work with such mega peptide pools, including ELISPOT, ICS, and the AID tests.

## Materials and Methods

### Peripheral Blood Mononuclear Cells

PBMC from healthy adult human donors were from CTL’s ePBMC library (CTL, Shaker Heights, OH, USA). The PBMC had been collected by HemaCare Blood Donor Center (Van Nuys, CA) under HemaCare’s IRB and sold to CTL identifying donors by code only while concealing the subjects’ identities. The donors’ age, sex, ethnicity, and HLA type are shown in **Supplementary Table 2**. HLA typing was contracted to the University of Oklahoma Health Science Center (Oklahoma City, OK). The ten subjects for this study were selected according to their HCMV-positive status. The frozen cells were thawed following an optimized protocol (20) resulting in viability > 90% for all samples. The PBMC were resuspended in CTL-Test™ Medium (from CTL), developed for low background and high signal performance in ELISPOT assays. The number of PBMC plated into the ImmunoSpot^®^ (ELISPOT) experiments was 3 × 10^5^ PBMC per well.

### Peptides and Antigens

Five hundred fifty-three nonamer peptides, spanning the entire amino acid (a.a.) sequence of the HCMV pp65 protein in steps of single a.a. were purchased from JPT (Berlin, Germany) as a FastTrack CD8+ T cell epitope library. These peptides were not further purified following their synthesis; however, individual peptides were analyzed by JPT using LC-MS. The average purity of these peptides was 56%. These peptides were delivered as lyophilized powder with each peptide present in a dedicated well of a 96-well plate, distributed across a total of six 96-well plates. Individual peptides were first dissolved in 50 μl DMSO, followed by addition of 200 μl of CTL-Test™ Medium so as to generate a “primary peptide stock solution” at 100 μg peptide/ml with 20% v/v DMSO. From each of these wells, a “secondary, 10X peptide stock solution” was prepared using a 96-well multichannel pipette, in which peptides were at a concentration of 2 μg/ml, with DMSO diluted to 0.4%. On the day of testing, 20 μl from each well was transferred “en block”, with a 96-well multi-channel pipette into pre-coated ImmunoSpot^®^ assay plates containing 80 μl CTL-Test™ Medium. Finally, 100 μl of PBMC (containing 3 × 10^5^ cells) in CTL-Test™ Medium was added resulting in a test peptide concentration of 0.2 μg/ml with DMSO present at 0.04% v/v.

UV-inactivated entire HCMV virions (HCMV Grade 2 antigen from CTL) at 10 μg/ml was used to recall HCMV-specific CD4 cells. CPI (from CTL) was used as a positive control because, unlike CEF peptides, CPI elicits T cell recall responses in all healthy donors (21). CPI is a combination of protein antigens derived from CMV, influenza and parainfluenza viruses, and was used at a final concentration of 6 μg/ml in ImmunoSpot^®^ assays.

### Human IFN-γ ImmunoSpot^®^ Assays

Single-color enzymatic ImmunoSpot^®^ kits from CTL were used for the detection of antigen-induced IFNγ-producing CD8+ T cells. Peptides or pp65 were plated at the above specified concentrations into capture antibody-precoated assay plates in a volume of 100 μl per well. These plates with the antigen were stored in a CO_2_ incubator for less than 1 h until the PBMC were thawed and ready for plating. The PBMC were added at 3 × 10^5^ cells/well in 100 μl CTL-Test™ Medium followed by a 24 h activation culture at 37°C and 9% CO_2_. Thereafter the cells were removed, IFNγ detection antibody was added, and the plate-bound cytokine was visualized by enzyme-catalyzed substrate precipitation. After washing, the plates were air-dried prior to scanning and counting of spot forming units (SFU). ELISPOT plates were analyzed using an ImmunoSpot^®^ S6 Reader, by CTL. For each well, SFU were automatically calculated by the ImmunoSpot^®^ Software using its Autogate™ function (22). The data are expressed as SFU per 3 × 10^5^ PBMC, whereby each SFU corresponds to the cytokine footprint of an individual IFNγ-producing T cell (23).

### Statistical Analysis

As SFU counts follow Gaussian (normal) distribution among replicate wells, the use of parametric statistics is appropriate to identify positive and negative responses, respectively (24). The 553 individual peptides of the pp65 nonamer peptide library were tested in single wells. For these peptides, the threshold for a positive response was set at SFU counts exceeding 3 SD of the mean SFU count detected in 18 replicate media control wells, the latter defining the background noise of the test system. This cut off criterion for weak (cryptic) responses renders the likelihood for false positive results at 0.3%. Dominant responses were defined by exceeding 10 SD, and subdominant responses between 5 and 10 SD of the negative control. Simple linear regressions were preformed to examine relationships between variables.

### HLA-Binding Predictions

We assessed peptide-HLA I presentation by predicting peptide-HLA I binding using HLA I allele-specific profile motif matrices (25). We considered that a given peptide binds to a specific HLA I molecule when its binding score ranks within the top 3% percentile of the binding scores computed for 1,000 random 9-mer peptides (average amino acid composition of proteins in the SwissProt database). Peptide binding to experimentally defined HLA-A*02:01 restricted epitopes was predicted using netMHCIpan (25) an IEDB analysis resource (12), reporting percentile binding score. The lower the percentile binding score the better the binding. We selected netMHCIpan because it is the NIH’s official recommended site that was created based on the consensus of earlier epitope prediction algorithms. Moreover, netMHCIpan allows to target more MHC I molecules for peptide binding predictions than any other method.

### Previously Defined Epitopes

Epitope data for HLA-A2-restricted CD8+ T cell recognition was obtained from IEDB (12) with the following search settings: positive response only; host human; MHC I allele, HLA-A*0201, source species HCVM, source antigen: pp65. Only peptides 9 a.a. long were considered.

## Results and Discussion

### Experimental Design

Systematic CD8+ T cell epitope mapping for the HCMV pp65 protein requires 553 nonamer peptides to be tested individually on PBMC of single subjects. Due to the magnitude of this scope, such data have not been reported so far except for a recent feasibility study from our own group (17). We took advantage of the fact that ImmunoSpot^®^ assays require as few as 300,000 PBMC per antigen stimulation condition, and that these assays lend themselves to high-throughput testing and analysis. Utilizing only 173 million PBMC per subject, we therefore could test individually 553 nonamer HCMV pp65 peptides, along with 18 negative control replicate wells to establish the background noise, and the CPI positive control in triplicate.

Nonamer peptides were selected because the peptide binding groove of HLA class I molecules accommodates peptides 8–11 amino acid (aa) in length, with the most common peptide size being 9 aa residues (3). In contrast, HLA class II molecules present longer peptides (26). As such, the usage of nonamer peptides in our assays permitted preferential activation of CD8+ T cells (Some nonamer peptides can bind to HLA II molecules but there are only few CD4 T cell epitopes known that comprise of nonamers). Moreover, because the peptide binding groove of HLA class I molecules is closed on both ends (3), it is intolerant for frame shifts. The peptide library was designed therefore to walk the pp65 protein in steps of single a.a. with each nonamer peptide overlapping by 8 a.a. with the previous one (**Supplementary Figure 1**). Importantly, this approach enabled systematic coverage of every possible CD8+ T cell epitope within the pp65 antigen. By utilizing similar peptide libraries that are one or two amino acids shorter or longer than nine we possibly may have gained even more high-resolution data on epitope-reactive CD8+ T cells but for feasibility reasons such comparisons have been deferred.

To reduce assay variables, all peptides used in this study were from the same vendor, and were synthetized, stored, dissolved, and tested in the same way. Moreover, all the peptides were tested on each PBMC donor in a single experiment, which rendered the peptides the only assay variable.

Standard IFNγ ImmunoSpot^®^ assays with 24 h antigen exposure of PBMC were performed; a time period required for blast transformation and CD8+ T cell activation-driven IFNγ secretion to occur, but too short to permit CD8+ T cell proliferation or differentiation during the cell culture. Thus, we measured at single-cell resolution the frequencies of antigen-specific IFNγ-producing CD8+ T cells as they occurred *in vivo* at isolation of the PBMC. This approach, therefore permitted us to firmly measure within each PBMC sample the number of CD8+ T cells responding to each peptide, and thus to compare the frequencies of peptide-reactive CD8+ T cells to establish epitope hierarchies for each donor. Moreover, adding up all peptide-induced IFNγ SFU permits one to assess the cumulative magnitude of the antigen-specific CD8+ T cell population in each donor, in turn allowing for determination of the relative percentage of antigen-specific CD8+ T cells targeting individual epitopes in each test subject.

Such actual measurements of the epitope-specific CD8+ T cells were then compared to a) the recognition of published HLA-A^*^02:01-restricted epitopes, and b) epitope predictions not only for HLA-A^*^02:01, an allele that all 10 test subjects shared by design, but also for all other class I molecules expressed by the test subjects.

Epitope scans of this type do not permit to define the activation/differentiation states/lineages of the CD8+ T cells that recognize them. As most functional CD8+ memory/effector T cells secrete IFN-γ, however, screening for IFN-γ should be well suited for detecting epitopes that are targeted by CD8+ T cells (19). Once the number of possible peptides has been narrowed from hundreds to a couple per donor, it becomes possible to narrow in on such peptide-reactive CD8+ T cell populations, e.g.by studying their phenotype by multimers, or by testing their (co-) secretion of other cytokines and effector molecules by multicolor FluoroSpot (27).

### Highly Variable HCMV pp65 Epitope Recognition Patterns in HLA-A^*^02:01 Positive Subjects

Eighteen replicate wells containing media alone were included for all 10 individuals in our HCMV positive, HLA-A^*^02:01 positive cohort in order to firmly establish the background noise of the respective PBMC. The mean and standard deviation (SD) of this negative control is shown for all subjects in **Table 1**, also specifying the cut off values used for analyzing the peptide-induced SFU counts. The 533 individual pp65 nonamer peptides were also tested on each subjects’ PBMC, and the resulting peptide-induced SFU-counts graded: peptides triggering SFU counts larger than 3 and less or equal to 5 SD over the medium control were considered weakly positive or cryptic (highlighted in beige in **Table 1**). Of note, with the mean plus 3 SD definition utilized in this study, the chance for a datapoint being false positive was negligible, less than 0.3%. Peptides triggering SFU counts more than 5 and less than or equal 10 SD over the medium background (highlighted in yellow) were labeled subdominant, and peptides eliciting SFU counts exceeding 10 SD over the medium control were called dominant (and are labeled in orange in **Table 1**). We also introduced a fourth category for peptides that recalled CD8+ T cells in frequencies exceeding 100 SFU/300,000 PBMC, calling them super-dominant epitopes (shown in red in **Table 1**).

**Table 1:** HCMV ppp65 epitope recognition by CD8+ T cells in HCMV positive, HLA-A*02:01 positive subjects.

Peptides Tested		Individual Subjects' CD8+ T Cell Response (SFU per 300,000 PBMC)									
Peptide Name	Sequence	ID 1	ID 2	ID 3	ID 4	ID 5	ID 6	ID 7	ID 8	ID 9	ID 10
pp65:018‐026	ISGHVLKAV	0	2	2	0	7	1	72	11	0	6
pp65:030‐038	GDTPVLPHE	0	2	3	1	2	2	5	9	1	32
pp65:065‐073	STPCHRGDN	16	0	0	44	0	1	1	5	0	1
pp65:070‐078	RGDNQLQVQ	2	0	2	2	0	2	84	28	2	5
pp65:095‐103	HNPTGRSIC	15	0	20	0	0	1	2	10	1	1
pp65:097‐105	PTGRSICPS	0	0	41	1	21	0	9	5	0	2
pp65:103‐121	CPSQEPMSI	15	0	6	2	1	0	5	11	2	7
pp65:106‐114	QEPMSIYVY	14	0	16	5	0	0	2	13	1	3
pp65:107‐108	EPMSIYVYA	0	0	17	2	2	0	2	403	1	1
pp65:114‐121	YALPLKMLN	22	1	7	0	3	2	23	14	0	3
pp65:115‐123	ALPLKMLNI	13	0	5	7	2	0	6	22	6	2
pp65:116‐124	LPLKMLNIP	71	0	7	14	2	3	5	18	5	2
pp65:119‐127	KMLNIPSIN	5	0	6	102	2	1	21	10	0	0
pp65:139‐148	HRHLPVADA	13	1	1	18	1	1	6	9	5	3
pp65:141‐149	HLPVADAVI	7	0	1	0	26	0	5	8	0	3
pp65:142‐150	LPVADAVIH	11	0	2	10	0	0	0	6	10	1
pp65:144‐152	VADAVIHAS	1	2	5	0	44	1	2	3	3	6
pp65:149‐157	IHASGKQMW	0	0	525	1	2	0	1	2	2	2
pp65:151‐158	ASGKQMWQA	20	0	2	3	0	1	7	6	1	0
pp65:152‐160	SGKQMWQAR	23	0	9	7	0	1	10	9	3	1
pp65:155‐163	QMWQARLTV	1	1	7	1	10	2	33	13	5	0
pp65:175‐183	WKEPDVYYT	1	0	2	0	144	0	1	7	0	1
pp65:188‐196	FPTKDVALR	1	1	1	5	6	1	695	3	13	1
pp65:203‐211	ELVCSMENT	118	0	0	2	1	1	21	3	7	1
pp65:208‐216	MENTRATKM	1	1	71	7	5	1	0	14	11	3
pp65:221‐229	DQYVKVYLE	1	1	7	1	76	0	0	10	6	0
pp65:228‐236	LESFCEDVP	0	0	2	6	10	1	1	2	0	5
pp65:250‐258	VEEDLTMTR	3	0	3	6	13	3	2	2	2	1
pp65:251‐259	EEDLTMTRN	0	1	1	2	1	2	107	1	3	2
pp65:262‐270	PFMRPHERN	0	1	161	5	0	2	6	3	9	1
pp65:267‐275	HERNGFTVL	0	0	3	0	0	0	6	2	0	46
pp65:270‐278	NGFTVLCPK	0	2	10	0	1	0	5	9	7	310
pp65:273‐281	TVLCPKNMI	1	0	9	62	0	2	7	2	3	2
pp65:284‐292	PGKISHIML	11	0	0	11	2	7	6	7	10	18
pp65:320‐328	LMNGQQIFL	14	2	10	17	1	0	21	2	1	21
pp65:324‐332	QQIFLEVQA	343	0	5	3	3	0	6	5	1	8
pp65:325‐333	QIFLEVQAI	398	1	6	16	5	1	1	13	0	7
pp65:328‐336	LEVQAIRET	0	5	810	7	1	1	7	10	5	21
pp65:390‐398	EGAAQGDDD	0	0	5	56	0	0	7	13	2	6
pp65:395‐403	GDDDVWTSG	2	0	3	10	0	0	14	98	1	2
pp65:417‐425	TPRVTGGGA	0	0	3	32	0	1	10	2	558	2
pp65:418‐426	PRVTGGGAM	1	0	6	6	0	0	6	11	192	0
pp65:430‐438	STSAGRKRK	3	1	0	89	1	0	34	18	11	7
pp65:431‐439	TSAGRKRKS	0	0	8	9	0	1	92	17	3	2
pp65:465‐473	EEDTDEDSD	0	1	11	54	1	1	5	6	7	7
pp65:482‐490	FTWPPWQAG	0	21	1	14	1	1	3	3	10	3
pp65:492‐500	LARNLVPMV	21	2	5	6	0	0	2	1	5	0
pp65:495‐503	NLVPMVATV	60	303	1	100	97	148	287	674	14	318
pp65:503‐511	VQGQNLKYQ	2	1	512	1	0	1	5	3	5	0
pp65:511‐519	QEFFWDAND	2	1	6	9	0	0	8	17	1	95
pp65:512‐520	EFFWDANDI	0	28	5	10	1	1	8	13	17	61
pp65:513‐521	FFWDANDIY	1	25	5	6	0	1	2	8	8	100
pp65:514‐522	FWDANDIYR	0	2	2	1	0	1	10	13	10	44
pp65:521‐529	YRIFAELEG	2	1	80	5	0	0	28	7	1	5
pp65:524‐532	FAELEGVWQ	2	16	6	8	1	7	24	8	11	6
pp65:544‐552	QDALPGPCI	2	6	5	3	15	5	13	5	2	2
Negative Controls and Cut Off Values for Response Categories	$\bar{x}$	1.0	0.8	4.2	3.9	3.9	1.8	6.5	8.4	5.4	3.2
	σ	1.0	1.3	3.6	4.4	0.5	2.4	5.6	5.6	3.7	2.7
	$\bar{x}$*3σ	3.9	4.6	14.9	17.1	5.5	8.8	23.3	25.2	16.6	11.3
	$\bar{x}$*5σ	5.8	7.2	22.1	25.9	6.5	13.5	34.4	36.3	24.0	16.8
	$\bar{x}$*10σ	10.7	13.7	40.0	47.8	9.1	25.3	62.4	64.2	42.5	30.3
	>100 SFU	>100 SFU	>100 SFU	>100 SFU	>100 SFU	>100 SFU	>100 SFU	>100 SFU	>100 SFU	>100 SFU	>100 SFU

Ten subjects’ PBMC at 300,000 cells per well were challenged with a library of 553 nonamer peptides that systematically covered all possible CD8+ T cell epitopes of the HCMV ppp65 antigen. An IFN-γ ImmunoSpot assay was performed with the spot forming units (SFU) elicited by each peptide recorded. The mean and SD for 18 negative control media wells, and the cut-off values for the color- coded response categories are specified on the bottom of this Table. Only peptides that induced at least one dominant (orange) or super-dominant (red) recall response in at least one subject are listed. Peptides that have been described as HLA-A^*^02:01 restricted nonamer epitopes in the literature are highlighted in green.

**Table 1** lists peptides that induced at least one dominant or super-dominant recall response in at least one of the ten test subjects in our cohort. Only for these 56 select peptides of the 553 tested are SFU counts shown for the ten donors. Additionally, a color-coding system was utilized in **Table 1** to delineate whether the peptide recalled a super-, dominant, subdominant, cryptic, or no response in the test subject.

As revealed by the color code at a glance, epitope recognition followed highly individual patterns, that are closer dissected below.

### Multiple HCMV pp65 Epitopes Are Recognized in Each HLA-A^*^02:01 Positive Subject

As **Table 1** lists only super- and dominant recall responses (>10 SD over background), in **Supplementary Table 3** we list 58 additional peptides that induced subdominant recall responses (5–10 SD over the background) in at least one of the ten test subjects in our cohort. At a glance, the color code reveals that these peptides are also recognized in a highly individualized pattern. Peptides that recalled cryptic responses (3–5 SD over background) are not listed individually, but their number is specified for each test subject in **Table 2**, along with the number of subdominant, and dominant and super-dominant epitopes recognized in each donor. Adding up the number of epitopes in all four categories permits one to establish the cumulative number of CD8+ T cell epitopes recognized in each subject, which varied between 5 and 47 HCMV pp65-derived peptides in this cohort $\left(\bar{x}=29\pm 17\right)$. Thus, of the 553 peptides covering the 561 amino acid long pp65 protein, between 1% and 8% $\left(\bar{x}=5\%\right)$ of the peptides constituted a CD8+ T cell epitope in each individual, but for the entire cohort 114 peptides (21% of 553 peptides tested) were needed to recall all dominant (56 peptides) and subdominant (58 peptides) CD8+ T cell epitopes. These data draw attention to how critical it is for immune monitoring to hit the right peptides—those few super-dominant aleatory epitopes that the majority of CD8+ T cells target. The number of CD8+ T cells recognizing cryptic, subdominant and dominant epitopes, in spite of the numbers of such epitopes, does not add up in most subjects to the repertoire that is directed against the few super-dominant epitopes.

**Table 2:** HCMV ppp65 epitope category distribution in HCMV positive, HLA-A*02:01 positive subjects.

		Test Subjects' CD8+ T Cells Specific for Epitopes									
		ID 1	ID 2	ID 3	ID 4	ID 5	ID 6	ID 7	ID 8	ID 9	ID 10
**Cryptic Epitopes**											
	**Number**	11	23	32	24	6	2	14	3	8	21
	**Cum. SFU**	12.10	18.67	67.15	92.50	3.04	0.39	58.40	11.53	21.57	56.12
	**% of total SFU**	1.02%	4.41%	3.02%	13.54%	0.73%	0.24%	4.03%	1.04%	2.89%	5.09%
**Subdominant Epitopes**											
	**Number**	14	8	5	17	6	2	6	0	1	16
	**Cum. SFU**	45	35	63	230	14	21	194	0	7	132
	**% of total SFU**	4%	8%	3%	34%	3%	13%	13%	0%	1%	12%
**Dominant Epitopes**											
	**Number**	15	4	3	4	9	0	3	1	0	5
	**Cum. SFU**	281	72	147	193	263	0	178	73	0	221
	**% of total SFU**	24%	17%	7%	28%	63%	0%	12%	7%	0%	20%
**Super Dominant Epitopes**											
	**Number**	3	1	4	2	1	1	3	2	2	3
	**Cum. SFU**	847	298	1948	168	139	139	1019	1027	717	694
	**% of total SFU**	71%	70%	88%	25%	33%	86%	70%	92%	96%	63%
										
**Total Epitopes Recognized**		43	36	44	47	22	5	26	6	11	45
**Cumulative Spec. SFU**		1185	424	2226	683	418	161	1450	1111	746	1103

The number of cryptic, subdominant, dominant and super-dominant epitopes, as defined in the text, are shown for the individual test subjects, along with the sum of epitopes in each category (Total Epitopes Recognized) for each PBMC donor. The absolute number of CD8+ T cells targeting peptides in each category (Cum. SFU) is also shown. From the number of all pp65-specific CD8+ T cells detected in each subject (Cumulative Spec. SFU) the percentage of CD8+ T cells targeting peptides in each of the four response categories has been calculated (% of total SFU). The SFU counts shown are after subtracting the mean + 3 SD specificity cut off value. Because SFU counts for the cryptic category frequently were at the cut-off value, or barely exceeded it, they shown with two decimal places.

In the feasibility study for this paper (**Tables 1** and **2** of our publication (17) we studied HCMV negative subjects, finding no significant HCMV peptide-triggered IFN-γ spot formation. Confirming this notion, in a detailed study dedicated to chance cross-reactivity in various antigenic systems, we also did not find evidence for such (28). These data suggest that chance cross-reactivity does not play a role in the dominant and super-dominant HCMV responses we report here.

### The Majority of the pp65-Specific CD8+ T Cell Repertoire Targets Super-Dominant Epitopes

As T cells recognize processed peptides of antigens there is no reason to assume that a T cell specific for one peptide of the antigen will contribute differently to host defense than T cells recognizing another. Immune monitoring therefore needs to assess all antigen-specific CD8+ T cells irrespective of their fine specificity. In our systematic assessment of CD8+ T cell immunity to pp65, we defined this number as the sum of all SFU counts elicited by the individual epitopes in a subject. This cumulative number of pp65-specific CD8+ T cells is shown for each subject in **Table 2** as “Cum. Spec. SFU”. From this number, one can calculate what percentage of the pp65-specific CD8 + T cells occurs in each of the four response categories. As seen in **Table 2**, although the number of super-dominant epitopes was low in each subject (between 4 and 1), in eight of ten donors the majority of pp65-specific CD8+ T cells targeted these super-dominant epitopes. The percentage of CD8+ T cells specific for individual dominant and super-dominant epitopes is shown in **S. Table 4**.

### CD8+ T Cells Target pp65 Epitopes in an Aleatory Manner

The data in **Table 1** and **Supplementary Table 3** show that each subject in our cohort displayed a unique CD8+ T cell epitope recognition pattern. This might come as a surprise, as all these subjects were HLA-A^*^02:01 positive, and one might have expected that among the epitopes recognized there should be at least a shared subset, those restricted by the HLA-A^*^02:01 allele. To narrow our investigation on such HLA-A^*^02:01-restricted epitopes, we searched the IEDB database for HLA-A^*^02:01-restricted nonamer epitopes identifying 31 that have been experimentally verified so far: these are listed in **Supplementary Table 4** with the corresponding reference citations. With the exception of the epitope, pp65_495-503_, of all these 31 previously defined HLA-A^*^02:01-restricted epitopes five peptides recalled super-dominant CD8+ T cells responses in only two of the 10 test subjects, while seven additional peptides triggered occasional dominant recall responses. The rest of the 31 previously defined peptides elicited sporadic subdominant (n=4), cryptic (n=6) or no recall responses (n=8) at all. Importantly, donors who did not respond strongly or at all to these previously defined epitopes responded vigorously to other peptides of pp65 (**Table 1**). These previously defined HLA-A^*^02:01—restricted peptides were therefore also targeted in a dice like, aleatory manner in HLA-A^*^02:01 positive subjects.

Only one previously defined HLA-A^*^02:01—restricted epitope, pp65_495-503_, induced a dominant, or super-dominant recall response in eight of 10 subjects in our cohort (**Table 1** and **Supplementary Table 1**). However, this peptide was not targeted in two donors (ID3# and ID#9) who exhibited responses to other pp65-derived peptides in a super-dominant fashion. Intrigued by this finding, we tested 42 additional (52 in total) HCMV positive, HLA-A^*^02:01 positive subjects for their recall response to the pp65_495-503_ peptide. As shown in **Supplementary Figure 2**, the numbers of CD8+ T cells responding to the pp65_495-503_ peptide did not correlate (r^2^ = 0.01) to the numbers of T cells recalled by inactivated HCMV virus; which primarily activates HCMV-specific CD4+ T cells. Even though all these subjects have developed T cell immunity to HCMV, about one fourth of them either did not respond to the pp65_495-503_ peptide, or displayed a low frequency of pp65_495-503_-specific CD8+ T cells. This finding is consistent with the notion that the CD8+ T cell response to pp65_495-503_ peptide is also aleatory. Interestingly, although the HLA-A^*^02:01 restriction element was shared by all test subjects in our cohort, and despite the pp65_495-503_ peptide being displayed *in vivo via* natural processing and presentation, in some individuals this epitope triggered a super-dominant CD8+ T cell response while in other subjects it did not induce a detectable respond at all. Moreover, in yet other donors, the magnitude of the CD8+T cell response induced by this peptide was anywhere between these two extremes. However, the higher prevalence of recognition for this epitope (pp65_495-503_) compared to other previously defined HLA-A^*^02:01-restricted epitopes might have an unexpected reason: in addition to HLA-A^*^02:01, pp65_495-503_ received a top binding score for several additional HLA-class I alleles (see next).

### HLA Binding Scores Are Unreliable Predictors of Actual CD8+ T Cell Epitope Utilization

The participation of the other HLA class I alleles, beyond the shared HLA-A^*^02:01 restriction element, might explain the highly individual CD8+ T cell response pattern observed in our cohort. Based on extensive knowledge on the peptide binding properties of individual HLA alleles, reference search engines have been established that permit in silico predictions of which peptides fit the binding criteria of a given allele, and moreover, the strength of peptide binding can also be ranked. It has been widely anticipated that such in silico models will suffice to predict epitope utilization. In particular, when there is the need to select one or a few candidate epitopes, e.g. for multimer analysis, it is tempting to pick peptides that have the highest predicted binding score for the HLA allele of interest. In the following we address the validity of such an approach from three angles.

In the first two approaches we focused on predictions for HLA-A^*^02:01, the most studied HLA allele that is shared by all subjects in our cohort. We introduced into the netMHCIpan (25) search engine of the IEDB analysis resource (12) the individual sequences of our pp65 nonamer peptide library, resulting in the predicted pp65 ranking shown in **Table 3** (in which only the top 30 predicted peptides of 553 are shown). In Approach 1, we compared this *in silico* predicted epitope hierarchy for pp65 with the actual peptide recognition we detected in our cohort. As can be seen in **Table 3**, pp65_495-503_ ranked as the top binder, and indeed induced a CD8+ T cell response in the majority of our HLA-A^*^02:01 positive cohort (albeit in an aleatory manner, see above). Most of the other predicted peptides with high HLA-A^*^02:01 binding scores recalled CD8+T cells in low frequencies, and in an aleatory manner with each of these predicted peptides eliciting SFU in only one or two of the 10 test subjects. Except for the pp65_495-503_ peptide, none of the super-dominant, and few of the dominant responses were recalled by these top 30 HLA-A^*^02:01 binding peptides (compare with **Table 1**). One might rightfully argue that this is because those dominant peptides were restricted by, and are binders of alternative class I molecules present in our cohort. We will address this hypothesis below, in Approach 3.

**Table 3:** CD8+ T cell recognition of predicted high HLA-A*02:01 -binding peptides.

Peptide Tested			Individual Subjects' CD8+ T Cell Response (SFU per 300,000 PBMC)									
pp65 Rank	Percentile Binding Score	Peptide Name	ID 1	ID 2	ID 3	ID 4	ID 5	ID 6	ID 7	ID 8	ID 9	ID 10
1	0.06	pp65:495‐503	60	303	1	100	97	148	287	674	14	318
2	0.07	pp65:522‐530	5	6	1	9	0	8	5	11	8	10
3	0.11	pp65:040‐048	0	1	0	7	3	0	5	13	2	1
4	0.15	pp65:120‐128	8	0	0	2	2	0	9	15	3	8
5	0.15	pp65:340‐348	6	7	5	6	0	2	1	2	5	21
6	0.17	pp65:320‐328	14	2	3	17	1	0	21	2	1	21
7	0.17	pp65:347‐355	0	0	10	23	0	0	3	14	1	3
8	0.19	pp65:349‐357	1	1	13	15	1	0	1	9	1	6
9	0.23	pp65:014‐022	1	1	15	3	5	0	3	24	8	8
10	0.33	pp65;218‐226	0	0	7	2	0	0	6	25	5	0
11	0.33	pp65:112‐120	0	0	0	3	2	1	6	5	17	1
12	0.38	pp65:155‐163	1	1	5	1	10	2	33	13	5	0
13	0.57	pp65:491‐499	1	0	5	2	1	2	7	2	7	3
14	0.61	pp65:290‐298	2	9	10	14	5	1	3	6	5	9
15	0.71	pp65:425‐433	2	0	10	2	1	1	8	7	1	2
16	0.76	pp65:082‐090	2	0	2	10	0	0	10	13	0	1
17	0.82	pp65:115‐123	13	0	1	7	2	0	6	22	6	2
18	0.82	pp65:286‐294	1	2	2	16	8	1	0	3	5	13
19	0.85	pp65:105‐113	2	0	2	2	0	0	2	8	1	1
20	1.4	pp65:312‐320	5	2	3	1	3	0	5	2	3	2
21	1.4	pp65:216‐224	0	0	1	2	1	2	22	7	5	2
22	1.4	pp65:274‐282	0	0	8	25	1	3	6	13	2	0
23	1.4	pp65:227‐235	0	0	3	5	0	2	5	6	5	2
24	1.5	pp65:318‐326	2	1	1	25	0	0	3	1	0	8
25	1.5	pp65:296‐304	1	0	2	8	2	0	9	11	1	9
26	1.6	pp65:110‐118	8	0	3	18	3	0	5	2	5	10
27	1.6	pp65:042‐050	1	0	0	6	3	1	5	5	0	3
28	1.6	pp65:054‐062	1	0	1	9	0	0	1	18	3	13
29	2	pp65:186‐194	2	2	0	1	1	2	5	5	18	7
30	2	pp65:319‐327	0	0	3	15	0	1	1	2	0	1
Negative Controls and Cut Off Values for Response Categories		$\bar{x}$	1.0	0.8	4.2	3.9	3.9	1.8	6.5	8.4	5.4	3.2
		σ	1.0	1.3	3.6	4.4	0.5	2.4	5.6	5.6	3.7	2.7
		$\bar{x}$*3σ	3.9	4.6	14.9	17.1	5.5	8.8	23.3	25.2	16.6	11.3
		$\bar{x}$*5σ	5.8	7.2	22.1	25.9	6.5	13.5	34.4	36.3	24.0	16.8
		$\bar{x}$*10σ	10.7	13.7	40.0	47.8	9.1	25.3	62.4	64.2	42.5	30.3
		>100 SFU	>100 SFU	>100 SFU	>100 SFU	>100 SFU	>100 SFU	>100 SFU	>100 SFU	>100 SFU	>100 SFU	>100 SFU

All 553 nonamer pp65 peptides in our library were run on the netMHCIpan search engine of the IEDB Analysis Resource for predicting their binding to the HLA-A^*^02:01 allele, resulting in “pp65 Rank” shown, with the top binder peptide ranked No. 1. The 30 highest ranking peptides are listed. Additionally, an Percentile Binding Score that compares a peptide’s binding relative to the binding scores computed for 1,000 random nonamer peptides, reporting the percentile binding score, is also listed. A lower percentile binding score denotes better peptide binding to the HLA-A^*^02:01 allele. Peptides that have been described as HLA-A^*^02:01 restricted nonamer epitopes in the literature are highlighted in green and the color-coded response categories defined in **Table 1** were applied.

In Approach 2, we looked up the predicted HLA-A^*^02:01 binding scores for those peptides that have been identified experimentally as HLA-A^*^02:01-restricted pp65 epitopes. In **Supplementary Table 1** these peptides have been listed according to their predicted HLA-A^*^02:01 binding ranking along with CD8+ T cell recall responses they induced in our HLA-A^*^02:01 positive cohort. With the exception of the pp65_495-503_ peptide, none of these peptides were among the predicted top 20 binders. Seeking for a correlation between the predicted HLA-A^*^02:01 binding of these peptides, and their actual immune dominance, these data are also represented graphically in **Supplementary Figure 3**. No significant correlation was seen. The fact, however, that these peptides were targeted by CD8+ T cells in HLA-A^*^02:01 positive subjects establishes that immune dominant epitopes do not need to rank high in peptide binding score. The score apparently needs to be just high enough to enable stable HLA allele binding.

Proteasome cleavage and TAP binding predictions can enhance CD8+ T cell epitope discrimination in silico as compared with peptide-MHC I binding predictions alone (29). These data suggest, however, that such refinements to epitope predictions might not suffice to improve the ability to foretell actually recognized epitopes. All 31 peptides in **Supplementary Table 1** are previously experimentally defined HLA-A^*^02:01-restricted epitopes. All of these peptides therefore passed proteasome and TAP selection. As there are no major known polymorphisms at the level of the proteasome or TAP binding, such are unlikely to contribute to the aleatory epitope recognition pattern observed for previously defined peptides. Therefore, rather than differences in antigen presentation, T cell repertoires and downstream repertoire selection processes are likely to explain the highly individualized epitope hierarchies seen in individuals.

**Figure 1:**
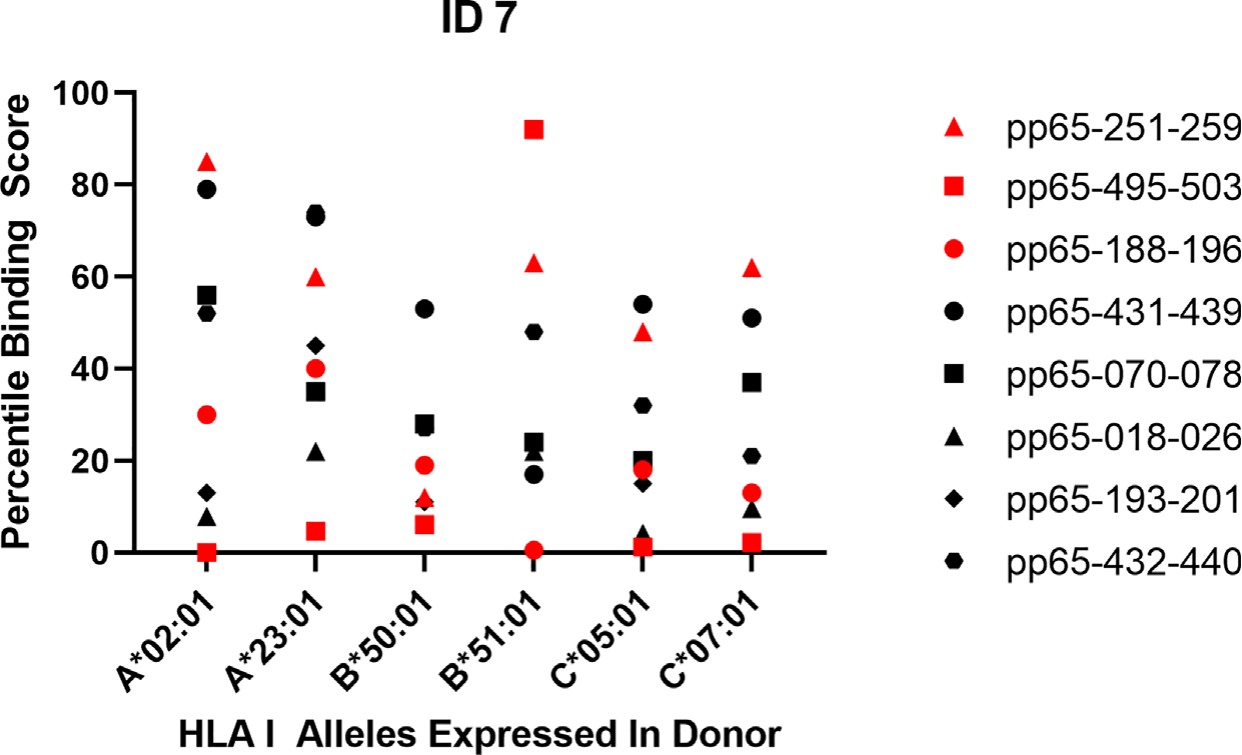
Predicted vs. actual pp65 epitope recognition by CD8+ T cells. Data are shown for subject ID 7 expressing the specified HLA alleles and responding to the listed peptides. Super-dominant responses are shown as red data points, dominant responses in black and weaker responses are not represented. The raw data for the peptide-induced SFU counts are listed in **Table 1**. The corresponding Percentile Binding Score as established by the netMHCIpan search engine is shown comparing a peptide’s binding relative to the binding scores computed for 1,000 random nonamer peptides. A lower percentile binding score denotes better peptide binding to the specified HLA allele.

In Approach 3, we matched binding predictions for all super-dominant and dominant epitopes detected in each of the 10 subjects with all HLA class I alleles expressed in the subject. The results shown for Subject ID 7 in **Figure 1** are fully representative for all other subjects in our cohort (see **Supplementary Figure 4**). For donor ID 7 three super-dominant epitopes were identified; these are represented by the red symbols. One is peptide 251–259 (the red triangle) that does not rank as a strong binder for any of the class I alleles present in this individual (a low “percentile binding score” on the Y axis of the graph means strong predicted binding). Based on the binding score the super-dominant status of this peptide could not have been predicted, and in this case the binding score also does not suggest what the restriction element might be. Peptide 495–503 (the red square) is also super dominant in this donor. It shows strong binding (a low score) for all class I alleles expressed in this individual, likely explaining its immunogenicity, but also suggesting that multiple restriction elements are involved (and that picking just one of them for a multimer is likely to underrepresent the 495–503-specific CD8+ T cell repertoire in this subject). Peptide 188–196 (the red dot) is also a super dominant epitope in donor ID 7. This peptide shows a high predicted binding score for the B*51:01 allele suggesting that as the restriction element. When designing B*51:01 multimers for immune monitoring for this donor, the 188–196 peptide would have been a hit—but why would one select one B allele over another B allele, neglecting all other loci and alleles, and if one did select top binders for each, most would be a miss. This erratic pattern carries through for all other dominant epitopes in this subject (the black symbols in **Figure 1**) and in all the other nine subjects we studied (**Supplementary Figure 4**). Few of the actually targeted CD8+ T cell epitopes ranked amongst the top binders for the class I alleles expressed in these respective subjects, and many super-dominant peptides ranked low. A binding score oriented in silico model would not have sufficed to predict the hierarchy of actual epitope recognition.

All three of our above approaches suggest that, at least in the case of CD8+ T cell immunity induced by HCMV infection against its pp65 antigen, in silico predicted high binding scores for a specific HLA class I allele neither predict whether those peptides will indeed induce a CD8+ T cell response, nor the magnitude of it. This finding raises the question how generalizable it is. Mei et al.’s recent report (8) suggests that it may be generalizable as they came to the same conclusion studying the prediction performance of databases containing 21,101 experimentally verified epitopes across 19 HLA class I alleles.

## Concluding Remarks

The scope of this study was to experimentally query whether CD8+ T cell epitope recognition for a prototypic foreign antigen follows immune dominance patterns that permit to predict the peptides recognized so immune monitoring can focus on them. We studied HCMV pp65 antigen recognition by CD8+ T cells in HCMV infected subjects at the highest possible resolution, testing every potential epitope and measuring the numbers of all epitope-specific CD8+ T cells. Our data show that fixed epitope hierarchies do not exist even in an HLA-A^*^02:01 allele matched cohort. Instead, different super-dominant and dominant epitopes were targeted by the individual test subjects (**Table 1**). Previously defined epitopes, and peptides predicted to be high HLA-A^*^02:01 binders also were also targeted in some, but not other individuals, if at all (**Supplementary Table 1** and **Table 3**). If generalizable, the notion of such unpredictable, aleatory epitope recognition patterns in individuals makes it obsolete for CD8+ T cell immune monitoring to rely on testing a select few predicted or previously defined peptides. Rather, comprehensive CD8+T cell immune monitoring must be all inclusive, accommodating all potential epitopes on all restriction elements of each test subject. Such can be accomplished by brute force epitope mapping, as we did here, or by the use of mega peptide pools.

Brute force epitope mapping might be required as the first step towards high definition CD8+ T cell monitoring, permitting the personalization od multimers. It identifies the few individually variable super dominant epitopes in an individual against which the vast majority of CD8+ T cells are directed (**Table 2**). In a second step, the effector lineages/differentiation states of these CD8+ T cells then can be closer characterized either by studying either their phenotype (19), and/or functions (27), whereby both steps can be pursued sequentially testing aliquots of cryopreserved PBMC. For the first step in this study we used 300,000 PBMC per well so as gain a high-resolution picture of epitope utilization detecting even low frequency CD8+ T cells. For testing 553 individual peptides at 300,000 PBMC we needed 173 million PBMC that can be readily obtained from healthy donors, but less so from diseased individuals and children. When epitope mapping aims at detecting super-dominant and dominant peptides only, this can be accomplished with substantially less PBMC. As the numbers of peptide-triggered SFU counts vs. the number of PBMC plated per well show a linear relationship between one million and 100,000 PBMC per well in regular 96-well plates (30), the results provided here could have been obtained with 58 million PBMC testing each peptide individually at 100,000 PBMC/well (however, no longer reliably detecting all subdominant and cryptic epitopes, however), and with e.g. one-fifth of that (12 million PBMC) if the peptides are tested in matrices (31). ELISPOT assay can be further miniaturized to 384-well format requiring one third of PBMC per well compared to the 96-well format (the membrane surface of the 384-well plate is one-third that of the 96-well plate), thus, only four million PBMC could suffice for the agnostic mapping of super dominant and dominant epitopes for an antigen the size of pp65 (30).

The highly individualized nature of CD8+ T cell epitope recognition might also be accommodated by the agnostic use of mega peptide pools. Those presently available consist of 15-mer peptides that walk the protein sequence with gaps of four amino acids and contain up to 200 peptides per pool. In a feasibility study towards this publication (17) we tested such a pp65 peptide pool (15-mers, 4 a.a. gaps, 138 peptides) along with the 9-mer epiScan. The number of peptide pool-triggered IFN-γ producing (CD4+ and CD8+) T cells approximated the number of all 9-mer peptide-induced CD8+ T cells when the latter were added up. However, for several theoretical reasons we are reluctant to propose the use of such 15-mer peptide pools for CD8+ T cell immune monitoring. First, 15-mer peptides are ideal for binding to HLA class II molecules stimulating CD4+ T cells, but they cannot directly bind to class I molecules whose peptide-binding grove is closed on both ends thus not only prevents the direct accommodation of peptides this long. One possibility for a 15-mer peptide to provide a CD8+ T cell epitope is that the peptide is cross presented —a process that is dependent on a subtype of dendritic cells that is too rare in PBMC (32) to be a prevalent APC type in *in vitro* recall assays. Another possibility is that peptidases present in the PBMC culture trim the 15-mer peptide to a length that can be accommodated by class I molecules, or that there are shorter byproducts of the 15-mer peptide synthesis present that can bind directly, or both. Thus, to the extent CD8+ T cells are recalled by 15-mer peptide pools, such recall can be expected to occur under highly suboptimal conditions. In addition, covering the protein sequence in steps of 11 a.a. leaves considerable gaps in CD8+ T cell epitope coverage which is of additional concern as the closed peptide binding grove of class I molecules renders peptide binding intolerant to frame shifts in the anchor residues of an epitope. Mega peptide pools ideal for CD8+ T cell monitoring would consist of 9-mer peptides that cover the protein sequence in steps of single amino acids.

While in silico epitope ranking may have limited value in predicting immune dominant peptides, it should be helpful for narrowing in on the subset of peptides on an antigen that has sufficient HLA-allele binding affinity to constitute an epitope. As it is impractical to tailor a multitude of variable peptides to each individual’s HLA-type, it might be more realistic for immune monitoring to develop rules for identifying peptides that do not bind to any HLA class I allele, so as to exclude those peptides from testing. Being able to narrow in on peptides should be helpful, as the ultimate goal of immune monitoring is to assess the CD8+ T cell response to the entire proteome of complex antigenic systems, such as all protein antigens of viruses and tumors.

## Data Availability Statement

The raw data supporting the conclusions of this article will be made available by the authors, without undue reservation.

## Ethics Statement

Ethical review and approval were not required for the study on human participants in accordance with the local legislation and institutional requirements. The patients/participants provided their written informed consent to participate in this study.

## Author Contributions

Experiments were designed by AL, PL and PR. Experimental data were generated by AL with TZ contributing. This publication serves as part of AL’s doctoral thesis to be submitted to the Universidad Complutense de Madrid, Madrid, Spain. All authors contributed to the article and approved the submitted version.

## Funding

This study was funded by the R&D budget of Cellular Technology Limited.

## Conflict of Interest

AL, TZ, and PL are employees of Cellular Technology Limited (CTL), a company that specializes on immune monitoring *via* ELISPOT testing, producing high-throughput-suitable readers, test kits, and offering GLP-compliant contract research.

The remaining author declares that the research was conducted in the absence of any commercial or financial relationships that could be construed as a potential conflict of interest.

## Supplementary Material

Supplementary Table 1 Actual recognition of previously identified HLA-A*02:01-restricted nonamer epitopes in our HCMV positive, HLA-A*02:01 positive cohort. The listed peptides have been identified in the IEDB database as HLA-A*02:01-restricted epitopes and the corresponding publications are specified. All 553 nonamer pp65 peptides in our library were run on IEDB’s netMHCIpan search engine for predicting their binding to the HLA-A*02:01 allele, resulting in the “pp65 Rank” shown, with the top binding peptide ranked No. 1. The corresponding Percentile Binding Score is shown comparing each peptide’s binding relative to the binding scores computed for 1,000 random nonamer peptides. A lower percentile binding score denotes better peptide binding to HLA-A*02:01. Otherwise, the legend to **Table 1** applies. Following references cited in the table refer to the bibliography (17, 33–50).

Supplementary Table 2 HLA class I allotypes and other characteristics of human subjects tested in this study.

Supplementary Table 3 Subdominant pp65 epitopes recognized in our cohort of ten HCMV positive, HLA-A*02:01 positive subjects. Peptides that elicited CD8+ T cell recall responses between 5 and 10 SD over the media background in at least one of the test subjects are highlighted in yellow. Cryptic recall responses (SD 3–5 over background) triggered by these peptides are highlighted in beige. Peptides that elicited SFU counts exceeding 10 SD over background are not shown in this table, as they are listed in **Table 1**. Peptides that only elicited cryptic recall responses (mean plus 3–5 SD) are not shown here, but are summarized in **Table 2**. Otherwise the legend to **Table 1** applies.

Supplementary Table 4 Percentage of pp65-specific CD8+ T cells targeting individual epitopes. The total number of pp65-specific CD8+ T cells was calculated from the sum of SFU triggered by all epitopes in each subject as detailed in **Table 2**. The percentages of Cumulative Specific SFU counts elicited by individual peptides in each test subjects are shown. For the corresponding absolute SFU counts see **Table 1**. Otherwise, the legend to **Table 1** applies.

Supplementary Figure 1 Schematic representation of brute force CD8+ T cell epitope mapping. The amino acid sequence of the protein, illustrated on the top, is covered with nonamer peptides that walk the sequence in steps of single amino acids.

Supplementary Figure 2 Frequency of HCMV pp65_495-503_ peptide-specific CD8+ T cells vs. HCMV grade 2 antigen-reactive T cells. Fifty-two subjects were selected from the ePBMC database for being HLA-A*02:01 positive and responding to HCMV Grade 2 antigen with more than 100 SFU/300,000 PBMC. Each of these subjects’ PBMC (represented by a dot) were re-tested in an ImmunoSpot assay for the numbers of SFU triggered by HCMV Grade 2 antigen (shown on the X axis), and the numbers of SFU elicited by the pp65_495-503_ peptide (shown on the Y axis). No significant relationship was found by analysis through a simple linear regression.

Supplementary Figure 3 HLA-A*02:01 binding ranking of previously defined HLA-A*02:01 restricted nonamer pp65 peptides vs. the SFU counts they induced in our cohort of HCMV positive, HLA-A*02:01 positive subjects. The numeric SFU data shown in **S. Table 1** are plotted relative to their Percentile Binding Score as established run on the netMHCIpan search engine for predicting their binding to the HLA-A*02:01 allele. No significant relationship was found by analysis through a simple linear regression.

Supplementary Figure 4 Predicted vs. actual pp65 epitope recognition by CD8+ T cells. Data are shown for the subjects specified in each panel. For each subject his/her HLA class I alleles are specified (in the case of homozygosity the allele is listed once). Peptides that induced super-dominant responses in that individual are shown as red data points, dominant responses in black and weaker responses are not represented. The raw data for the peptide-induced SFU counts are listed in **Table 1**. The IEBD Rank shown for each peptide and allele was established using the netMHCIpan search engine predicting the peptides’ binding score to the respective HLA allele, whereby a lower Percentile Binding Score binding score denotes better peptide binding.
