# Supplementary material for: Discordance Between the Predicted Versus the Actually Recognized CD8+ T Cell Epitopes of HCMV pp65 Antigen and Aleatory Epitope Dominance
Source: Front Immunol. 2021 Feb 9;11:618428. doi: 10.3389/fimmu.2020.618428 (PMC7900545; doi:10.3389/fimmu.2020.618428)
Supplement: Supplementary file 1 [file DataSheet_1.pdf]

Ref.	Peptides Tested				Inidiviudal Subjets' CD8+ T Cell Response (SFU per 300,000 PBMC)									
	Peptide Name	Epitope Sequence	Percentile Binding Score	pp65 Rank	ID 1	ID 2	ID 3	ID 4	ID 5	ID 6	ID 7	ID 8	ID 9	ID 10
**45,46,47,48**	pp65:495-503	NLVPMVATV	0.06	1	60	303	1	100	97	148	287	674	14	318
**36**	pp65:340-348	RQYDPVAAL	0.06	2	6	7	5	6	0	2	1	2	5	21
**33,40**	pp65:040-048	RLLQTGIHV	0.09	3	0	1	2	7	3	0	5	13	2	1
**33,39,40,41,42**	pp65:522-530	RIFAELEGV	0.11	5	5	6	0	9	0	8	5	11	8	10
**34,40**	pp65:320-328	LMNGQQIFL	0.15	4	14	2	10	17	1	0	21	2	1	21
**33**	pp65:218-226	VIGDQYVKV	0.23	7	0	0	10	3	2	1	6	5	17	1
**34,35**	pp65:155-163	QMWQARLTV	0.24	6	1	1	7	1	10	2	33	13	5	0
**43,44**	pp65:014-022	VLGPISGHV	0.24	9	1	1	10	3	5	0	3	24	8	8
**33,37,41,43,44,49,50**	pp65:120-128	MLNIPSINV	0.25	11	8	0	5	2	2	0	9	15	3	8
**36**	pp65:347-355	ALFFFDIDL	0.54	12	0	0	8	23	0	0	3	14	1	3
**40**	pp65:491-499	ILARNLVPM	0.74	13	1	0	5	2	1	2	7	2	7	3
**33**	pp65:425-433	AMAGASTSA	0.85	15	2	0	5	2	1	1	8	7	1	2
**37**	pp65:042-050	LQTGIHVRV	0.98	21	1	0	6	18	3	0	5	2	5	10
**33**	pp65:054-062	SLILVSQYT	1.5	23	1	0	1	6	3	1	5	5	0	3
**17**	pp65:325-333	QIFLEVQAI	1.6	27	398	1	6	16	5	1	1	13	0	7
**33**	pp65:312-320	GLSISGNLL	1.9	26	5	0	9	5	0	2	5	6	5	2
**33**	pp65:110-118	SIYVYALPL	2.2	28	8	0	5	9	0	0	1	18	3	13
**33**	pp65:227-235	YLESFCEDV	2.6	32	7	0	1	2	1	2	22	7	5	2
**37,38**	pp65:341-349	QYDPVAALF	3.3	36	1	9	9	28	0	0	1	9	1	2
**17**	pp65:324-332	QQIFLEVQA	4	43	343	0	5	3	3	0	6	5	1	8
**33**	pp65:519-527	DIYRIFAEL	4.3	49	1	0	7	0	2	1	14	3	1	1
**17**	pp65:141-149	HLPVADAVI	5.1	54	7	0	1	0	26	0	5	8	0	3
**17**	pp65:144-152	VADAVIHAS	11	80	1	2	5	0	44	1	2	3	3	6
**33**	pp65:509-517	KYQEFFWDA	12	92	0	2	2	2	0	3	11	2	2	7
**36**	pp65:345-353	VAALFFFDI	16	97	0	6	2	23	0	0	8	3	2	5
**17**	pp65:203-211	ELVCSMENT	23	163	118	0	0	2	1	1	21	3	7	1
**17**	pp65:221-229	DQYVKVYLE	25	229	1	1	7	1	76	0	0	10	6	0
**17**	pp65:116-124	LPLKMLNIP	51	360	71	0	7	14	2	3	5	18	5	2
**17**	pp65:417-425	TPRVTGGGA	63	378	0	0	3	32	0	1	10	2	558	2
**17**	pp65:418-426	PRVTGGGAM	73	394	1	0	6	6	0	0	6	11	192	0
**17**	pp65:097-105	PTGRSICPS	78	510	0	0	41	1	21	0	9	5	0	2
