# Supplementary material for: Discordance Between the Predicted Versus the Actually Recognized CD8+ T Cell Epitopes of HCMV pp65 Antigen and Aleatory Epitope Dominance
Source: Front Immunol. 2021 Feb 9;11:618428. doi: 10.3389/fimmu.2020.618428 (PMC7900545; doi:10.3389/fimmu.2020.618428)
Supplement: Supplementary Table 1 — Actual recognition of previously identified HLA-A*02:01-restricted nonamer epitopes in our HCMV positive, HLA-A*02:01 positive cohort. The listed peptides have been identified in the IEDB database as HLA-A*02:01-restricted epitopes and the corresponding publications are specified. All 553 nonamer pp65 peptides in our library were run on IEDB’s netMHCIpan search engine for predicting their binding to the HLA-A*02:01 allele, resulting in the “pp65 Rank” shown, with the top binding peptide ranked No. 1. The corresponding Percentile Binding Score is shown comparing each peptide’s binding relative to the binding scores computed for 1,000 random nonamer peptides. A lower percentile binding score denotes better peptide binding to HLA-A*02:01. Otherwise, the legend to Table 1 applies. Following references cited in the table refer to the bibliography (17, 33–50). [file Table_1.pdf]

Ref.	Peptides Tested				Inidiviudal Subjets' CD8+ T Cell Response (SFU per 300,000 PBMC)									
	Peptide Name	Epitope Sequence	Percentile Binding Score	pp65 Rank	ID 1	ID 2	ID 3	ID 4	ID 5	ID 6	ID 7	ID 8	ID 9	ID 10
45,46,47,48	pp65:495-503	NLVPMVATV	0.06	1	60	303	1	100	97	148	287	674	14	318
36	pp65:340-348	RQYDPVAAL	0.06	2	6	7	5	6	0	2	1	2	5	21
33,40	pp65:040-048	RLLQTGIHV	0.09	3	0	1	2	7	3	0	5	13	2	1
33,39,40,41,42	pp65:522-530	RIFAELEGV	0.11	5	5	6	0	9	0	8	5	11	8	10
34,40	pp65:320-328	LMNGQQIFL	0.15	4	14	2	10	17	1	0	21	2	1	21
33	pp65:218-226	VIGDQYVKV	0.23	7	0	0	10	3	2	1	6	5	17	1
34,35	pp65:155-163	QMWQARLTV	0.24	6	1	1	7	1	10	2	33	13	5	0
43,44	pp65:014-022	VLGPISGHV	0.24	9	1	1	10	3	5	0	3	24	8	8
33,37,41,43,44,49,50	pp65:120-128	MLNIPSINV	0.25	11	8	0	5	2	2	0	9	15	3	8
36	pp65:347-355	ALFFFDIDL	0.54	12	0	0	8	23	0	0	3	14	1	3
40	pp65:491-499	ILARNLVPM	0.74	13	1	0	5	2	1	2	7	2	7	3
33	pp65:425-433	AMAGASTSA	0.85	15	2	0	5	2	1	1	8	7	1	2
37	pp65:042-050	LQTGIHVRV	0.98	21	1	0	6	18	3	0	5	2	5	10
33	pp65:054-062	SLILVSQYT	1.5	23	1	0	1	6	3	1	5	5	0	3
17	pp65:325-333	QIFLEVQAI	1.6	27	398	1	6	16	5	1	1	13	0	7
33	pp65:312-320	GLSISGNLL	1.9	26	5	0	9	5	0	2	5	6	5	2
33	pp65:110-118	SIYVYALPL	2.2	28	8	0	5	9	0	0	1	18	3	13
33	pp65:227-235	YLESFCEDV	2.6	32	7	0	1	2	1	2	22	7	5	2
37,38	pp65:341-349	QYDPVAALF	3.3	36	1	9	9	28	0	0	1	9	1	2
17	pp65:324-332	QQIFLEVQA	4	43	343	0	5	3	3	0	6	5	1	8
33	pp65:519-527	DIYRIFAEL	4.3	49	1	0	7	0	2	1	14	3	1	1
17	pp65:141-149	HLPVADAVI	5.1	54	7	0	1	0	26	0	5	8	0	3
17	pp65:144-152	VADAVIHAS	11	80	1	2	5	0	44	1	2	3	3	6
33	pp65:509-517	KYQEFFWDA	12	92	0	2	2	2	0	3	11	2	2	7
36	pp65:345-353	VAALFFFDI	16	97	0	6	2	23	0	0	8	3	2	5
17	pp65:203-211	ELVCSMENT	23	163	118	0	0	2	1	1	21	3	7	1
17	pp65:221-229	DQYVKVYLE	25	229	1	1	7	1	76	0	0	10	6	0
17	pp65:116-124	LPLKMLNIP	51	360	71	0	7	14	2	3	5	18	5	2
17	pp65:417-425	TPRVTGGGA	63	378	0	0	3	32	0	1	10	2	558	2
17	pp65:418-426	PRVTGGGAM	73	394	1	0	6	6	0	0	6	11	192	0
17	pp65:097-105	PTGRSICPS	78	510	0	0	41	1	21	0	9	5	0	2
