# Supplementary material for: Discordance Between the Predicted Versus the Actually Recognized CD8+ T Cell Epitopes of HCMV pp65 Antigen and Aleatory Epitope Dominance
Source: Front Immunol. 2021 Feb 9;11:618428. doi: 10.3389/fimmu.2020.618428 (PMC7900545; doi:10.3389/fimmu.2020.618428)
Supplement: Supplementary Table 2 — HLA class I allotypes and other characteristics of human subjects tested in this study. [file Table_2.pdf]

Code	Ethnicity	Age	Gender	Blood Type	HLA A	HLA B	HLA C
ID 1	Hispanic/Latino	36	Male	O/Pos	A*02:01	B*40:08	C*03:04
					A*68:01	B*44:03	C*16:01
ID 2	Hispanic	30	Male	O/Pos	A*02:01	B*39:05	C*02:02
					A*24:02	B*51:01	C*07:02
ID 3	Hispanic	18	Male	A/Pos	A*02:01	B*07:02	C*04:01
					A*02:01	B*35:17	C*07:02
ID 4	Caucasian	25	Male	O/Pos	A*02:01	B*27:05	C*01:02
					A*23:01	B*58:01	C*07:01
ID 5	Hispanic	40	Male	O/Pos	A*02:01	B*39:05	C*07:02
					A*68:03	B*51:01	C*15:09
ID 6	Hispanic/Caucasian	39	Female	O/Pos	A*02:01	B*18:01	C*01:02
					A*25:01	B*56:01	C*12:03
ID 7	Hispanic	33	Female	O/Pos	A*02:01	B*50:01	C*05:01
					A*23:01	B*51:01	C*07:01
ID 8	Caucasian	41	Male	O/Pos	A*02:01	B*13:02	C*04:01
					A*23:01	B*44:03	C*06:02
ID 9	Caucasian	71	Male	O/Pos	A*02:01	B*07:24	C*07:01
					A*03:01	B*18:01	C*07:02
ID 10	Caucasian	62	Male	B/Pos	A*02:01	B*41:02	C*14:02
					A*66:01	B*51:01	C*17:01
