# Supplementary material for: Discordance Between the Predicted Versus the Actually Recognized CD8+ T Cell Epitopes of HCMV pp65 Antigen and Aleatory Epitope Dominance
Source: Front Immunol. 2021 Feb 9;11:618428. doi: 10.3389/fimmu.2020.618428 (PMC7900545; doi:10.3389/fimmu.2020.618428)
Supplement: Supplementary Table 3 — Subdominant pp65 epitopes recognized in our cohort of ten HCMV positive, HLA-A*02:01 positive subjects. Peptides that elicited CD8+ T cell recall responses between 5 and 10 SD over the media background in at least one of the test subjects are highlighted in yellow. Cryptic recall responses (SD 3–5 over background) triggered by these peptides are highlighted in beige. Peptides that elicited SFU counts exceeding 10 SD over background are not shown in this table, as they are listed in Table 1 . Peptides that only elicited cryptic recall responses (mean plus 3–5 SD) are not shown here, but are summarized in Table 2 . Otherwise the legend to Table 1 applies. [file Table_3.pdf]

Peptides Tested		Inidiviudal Subjets' CD8+ T Cell Response (SFU per 300,000 PBMC)									
Sequence	Peptide Name	ID 1	ID 2	ID 3	ID 4	ID 5	ID 6	ID 7	ID 8	ID 9	ID 10
pp65:011-019	MISVLGPIS	0	2	0	1	2	0	51	24	1	2
pp65:021-029	HVLKAVFSR	2	0	1	1	1	2	46	6	3	1
pp65:028-036	SRGDTPVLP	1	0	1	3	1	2	2	20	0	18
pp65:046-054	IHVRVSQPS	6	2	0	6	1	2	5	10	0	5
pp65:053-061	PSLILVSQY	1	1	5	31	1	0	52	8	1	3
pp65:066-074	TPCHRGDNQ	0	0	6	31	1	1	2	3	1	9
pp65:110-118	SIYVYALPL	8	0	5	9	0	0	1	18	3	13
pp65:117-125	PLKMLNIPS	7	1	5	7	1	2	2	2	1	5
pp65:120-128	MLNIPSINV	8	0	5	2	2	0	9	15	3	8
pp65:126-134	INVHHYPSA	0	0	5	8	1	5	6	5	3	18
pp65:134-142	AAERKHRHL	2	0	3	3	0	0	7	10	1	18
pp65:140-148	RHLPVADAV	6	0	2	9	2	1	2	11	5	7
pp65:162-170	TVSGLAWTR	7	1	7	9	2	2	2	5	8	3
pp65:174-182	QWKEPDVYY	10	0	15	2	1	0	2	5	5	2
pp65:193-201	VALRHVVCA	0	1	11	0	0	0	62	3	2	0
pp65:196-204	RHVVCAHEL	0	0	23	0	1	0	21	7	7	1
pp65:202-210	HELVCSMEN	0	0	1	0	8	2	9	10	2	7
pp65:209-217	ENTRATKMQ	1	0	5	0	7	5	2	3	5	3
pp65:210-218	NTRATKMQV	1	1	40	2	3	5	0	6	8	1
pp65:264-272	MRPHERNGF	3	0	2	6	8	0	9	3	1	2
pp65:277-285	PKNMIIKPG	2	0	9	32	0	0	1	10	2	0
pp65:285-293	GKISHIMLD	0	0	8	40	0	2	0	6	0	0
pp65:286-294	KISHIMLDV	1	2	3	16	8	1	0	3	5	13
pp65:289-297	HIMLDVAFT	0	2	3	26	1	0	3	10	3	0
pp65:290-298	IMLDVAFTS	2	9	2	14	5	1	3	6	5	9
pp65:293-302	DVAFTSHEH	1	0	3	8	0	1	6	5	2	18
pp65:301-309	HFGLLCPKS	2	0	1	18	0	0	7	1	0	20
pp65:304-312	LLCPKSIPG	1	0	6	8	1	5	3	8	2	21
pp65:306-314	CPKSIPGLS	1	1	5	15	1	1	10	7	1	18
pp65:311-319	PGLSISGNL	1	1	3	29	1	0	3	7	0	3
pp65;316-324	SGNLLMNGQ	0	6	8	2	1	2	2	9	3	29
pp65:332-340	AIRETVELR	6	0	3	7	1	0	5	5	7	17
pp65:335-343	ETVELRQYD	0	3	3	26	2	7	0	2	0	2
pp65:337-345	VELRQYDPV	0	0	14	31	0	0	2	7	11	3
pp65:340-348	RQYDPVAAL	6	7	5	6	0	2	1	2	5	21
pp65:341-349	QYDPVAALF	1	9	9	28	0	0	1	9	1	2
pp65:346-354	AALFFFDID	0	5	24	7	0	0	5	14	2	6
pp65:351-359	FDIDLLLQR	1	0	17	18	0	2	1	7	2	20
pp65:356-364	LLQRGPQYS	7	2	14	2	0	2	8	9	1	11
pp65:363-371	YSEHPTFTS	2	0	6	28	1	0	2	6	1	6
pp65:365-373	EHPTFTSQY	0	0	11	26	1	0	2	6	1	1
pp65:368-374	TFTSQYRIQ	10	1	26	8	0	7	9	14	13	15
pp65:380-388	EYRHTWDRH	1	2	9	28	0	1	9	3	5	9
pp65:383-391	HTWDRHDEG	0	0	25	13	1	0	16	3	2	2
pp65:393-401	AQGDDDVWT	6	0	1	1	0	2	9	5	1	2
pp65:412-420	TTERKTPRV	2	1	9	13	1	1	61	5	6	1
pp65:432-440	SAGRKRKSA	1	2	7	8	2	2	62	9	0	5
pp65:460-468	STVAPEEDT	2	8	6	29	9	0	30	20	10	6
pp65:466-474	EDTDEDSDN	1	0	5	29	0	7	2	5	3	10
pp65:472-480	SDNEIHNPA	0	0	5	6	1	23	3	8	5	13
pp65:477-485	HNPAVFTWP	0	8	5	3	1	1	0	7	5	5
pp65:486-494	PWQAGILAR	6	11	9	5	3	1	6	9	2	17
pp65:496-504	LVPMVATVQ	0	5	15	15	0	5	14	1	10	18
pp65:526-534	ELEGVWQPA	2	7	2	23	1	16	3	6	1	1
pp65:531-539	WQPAAQPKR	0	8	2	10	0	3	10	1	5	1
pp65:534-542	AAQPKRRRH	3	1	0	6	2	1	7	3	24	6
pp65:535-548	AQPKRRRHR	0	10	2	3	1	1	10	3	3	5
pp65:552-560	IASTPKKHR	1	9	2	18	0	1	14	6	14	3
Negative Controls and Cut Off Values For Response Categories	$\bar{x}$	1.0	0.8	4.2	3.9	3.9	1.8	6.5	8.4	5.4	3.2
	σ	1.0	1.3	3.6	4.4	0.5	2.4	5.6	5.6	3.7	2.7
	$\bar{x}$*3σ	3.9	4.6	14.9	17.1	5.5	8.8	23.3	25.2	16.6	11.3
	$\bar{x}$*5σ	5.8	7.2	22.1	25.9	6.5	13.5	34.4	36.3	24.0	16.8
	$\bar{x}$*10σ	10.7	13.7	40.0	47.8	9.1	25.3	62.4	64.2	42.5	30.3
	>100 SFU	>100	>100	>100	>100	>100	>100	>100	>100	>100	>100
