# Supplementary material for: Discordance Between the Predicted Versus the Actually Recognized CD8+ T Cell Epitopes of HCMV pp65 Antigen and Aleatory Epitope Dominance
Source: Front Immunol. 2021 Feb 9;11:618428. doi: 10.3389/fimmu.2020.618428 (PMC7900545; doi:10.3389/fimmu.2020.618428)
Supplement: Supplementary Table 4 — Percentage of pp65-specific CD8+ T cells targeting individual epitopes. The total number of pp65-specific CD8+ T cells was calculated from the sum of SFU triggered by all epitopes in each subject as detailed in Table 2 . The percentages of Cumulative Specific SFU counts elicited by individual peptides in each test subjects are shown. For the corresponding absolute SFU counts see Table 1 . Otherwise, the legend to Table 1 applies. [file Table_4.pdf]

Peptides Tested		Inidiviudal Subjets' CD8+ T Cell Response (SFU per 300,000 PBMC)									
Peptide Name	Sequence	ID 1	ID 2	ID 3	ID 4	ID 5	ID 6	ID 7	ID 8	ID 9	ID 10
pp65:018-026	ISGHVLKAV	0.00%	0.00%	0.00%	0.00%	0.37%	0.00%	3.41%	0.00%	0.00%	0.00%
pp65:030-038	GDTPVLPHE	0.00%	0.00%	0.00%	0.00%	0.00%	0.00%	0.00%	0.00%	0.00%	1.93%
pp65:065-073	STPCHRGDN	1.02%	0.00%	0.00%	4.20%	0.00%	0.00%	0.00%	0.00%	0.00%	0.00%
pp65:070-078	RGDNQLQVQ	0.00%	0.00%	0.00%	0.00%	0.00%	0.00%	4.24%	0.26%	0.00%	0.00%
pp65:095-103	HNPTGRSIC	0.94%	0.00%	0.23%	0.00%	0.00%	0.00%	0.00%	0.00%	0.00%	0.00%
pp65:097-105	PTGRSICPS	0.00%	0.00%	1.17%	0.00%	3.81%	0.00%	0.00%	0.00%	0.00%	0.00%
pp65:103-121	CPSQEPMSI	0.94%	0.00%	0.00%	0.00%	0.00%	0.00%	0.00%	0.00%	0.00%	0.00%
pp65:106-114	QEPMSIYVY	0.86%	0.00%	0.05%	0.00%	0.00%	0.00%	0.00%	0.00%	0.00%	0.00%
pp65:107-108	EPMSIYVYA	0.00%	0.00%	0.09%	0.00%	0.00%	0.00%	0.00%	34.16%	0.00%	0.00%
pp65:114-121	YALPLKMLN	1.53%	0.00%	0.00%	0.00%	0.00%	0.00%	0.00%	0.00%	0.00%	0.00%
pp65:115-123	ALPLKMLNI	0.77%	0.00%	0.00%	0.00%	0.00%	0.00%	0.00%	0.00%	0.00%	0.00%
pp65:116-124	LPLKMLNIP	5.68%	0.00%	0.00%	0.00%	0.00%	0.00%	0.00%	0.00%	0.00%	0.00%
pp65:119-127	KMLNIPSIN	0.09%	0.00%	0.00%	13.24%	0.00%	0.00%	0.00%	0.00%	0.00%	0.00%
pp65:139-148	HRHLPVADA	0.77%	0.00%	0.00%	0.14%	0.00%	0.00%	0.00%	0.00%	0.00%	0.00%
pp65:141-149	HLPVADAVI	0.26%	0.00%	0.00%	0.00%	5.04%	0.00%	0.00%	0.00%	0.00%	0.00%
pp65:142-150	LPVADAVIH	0.60%	0.00%	0.00%	0.00%	0.00%	0.00%	0.00%	0.00%	0.00%	0.00%
pp65:144-152	VADAVIHAS	0.00%	0.00%	0.00%	0.00%	9.46%	0.00%	0.00%	0.00%	0.00%	0.00%
pp65:149-157	IHASGKQMW	0.00%	0.00%	22.94%	0.00%	0.00%	0.00%	0.00%	0.00%	0.00%	0.00%
pp65:151-158	ASGKQMWQA	1.36%	0.00%	0.00%	0.00%	0.00%	0.00%	0.00%	0.00%	0.00%	0.00%
pp65:152-160	SGKQMWQAR	1.62%	0.00%	0.00%	0.00%	0.00%	0.00%	0.00%	0.00%	0.00%	0.00%
pp65:155-163	QMWQARLTV	0.00%	0.00%	0.00%	0.00%	1.11%	0.00%	0.68%	0.00%	0.00%	0.00%
pp65:175-183	WKEPDVYYT	0.00%	0.00%	0.00%	0.00%	34.03%	0.00%	0.00%	0.00%	0.00%	0.00%
pp65:188-196	FPTKDVALR	0.00%	0.00%	0.00%	0.00%	0.12%	0.00%	46.94%	0.00%	0.00%	0.00%
pp65:203-211	ELVCSMENT	9.66%	0.00%	0.00%	0.00%	0.00%	0.00%	0.00%	0.00%	0.00%	0.00%
pp65:208-216	MENTRATKM	0.00%	0.00%	2.52%	0.00%	0.00%	0.00%	0.00%	0.00%	0.00%	0.00%
pp65:221-229	DQYVKVYLE	0.00%	0.00%	0.00%	0.00%	17.32%	0.00%	0.00%	0.00%	0.00%	0.00%
pp65:228-236	LESFCEDVP	0.00%	0.00%	0.00%	0.00%	1.11%	0.00%	0.00%	0.00%	0.00%	0.00%
pp65:250-258	VEEDLTMTR	0.00%	0.00%	0.00%	0.00%	1.84%	0.00%	0.00%	0.00%	0.00%	0.00%
pp65:251-259	EEDLTMTRN	0.00%	0.00%	0.00%	0.00%	0.00%	0.00%	5.85%	0.00%	0.00%	0.00%
pp65:262-270	PFMRPHERN	0.00%	0.00%	6.57%	0.00%	0.00%	0.00%	0.00%	0.00%	0.00%	0.00%
pp65:267-275	HERNGFTVL	0.00%	0.00%	0.00%	0.00%	0.00%	0.00%	0.00%	0.00%	0.00%	3.23%
pp65:270-278	NGFTVLCPK	0.00%	0.00%	0.00%	0.00%	0.00%	0.00%	0.00%	0.00%	0.00%	27.84%
pp65:273-281	TVLCPKNMI	0.00%	0.00%	0.00%	7.00%	0.00%	0.00%	0.00%	0.00%	0.00%	0.00%
pp65:284-292	PGKISHIML	0.60%	0.00%	0.00%	0.00%	0.00%	0.00%	0.00%	0.00%	0.00%	0.62%
pp65:320-328	LMNGQQIFL	0.86%	0.00%	0.00%	0.00%	0.00%	0.00%	0.00%	0.00%	0.00%	0.90%
pp65:324-332	QQIFLEVQA	28.71%	0.00%	0.00%	0.00%	0.00%	0.00%	0.00%	0.00%	0.00%	0.00%
pp65:325-333	QIFLEVQAI	33.37%	0.00%	0.00%	0.00%	0.00%	0.00%	0.00%	0.00%	0.00%	0.00%
pp65:328-336	LEVQAIRET	0.00%	0.09%	35.77%	0.00%	0.00%	0.00%	0.00%	0.00%	0.00%	0.90%
pp65:390-398	EGAAQGDDD	0.00%	0.00%	0.00%	6.07%	0.00%	0.00%	0.00%	0.00%	0.00%	0.00%
pp65:395-403	GDDDVWTSG	0.00%	0.00%	0.00%	0.00%	0.00%	0.00%	0.00%	6.59%	0.00%	0.00%
pp65:417-425	TPRVTGGGA	0.00%	0.00%	0.00%	2.32%	0.00%	0.00%	0.00%	0.00%	73.07%	0.00%
pp65:418-426	PRVTGGGAM	0.00%	0.00%	0.00%	0.00%	0.00%	0.00%	0.00%	0.00%	23.68%	0.00%
pp65:430-438	STSAGRKRK	0.00%	0.00%	0.00%	11.22%	0.00%	0.00%	0.75%	0.00%	0.00%	0.00%
pp65:431-439	TSAGRKRKS	0.00%	0.00%	0.00%	0.00%	0.00%	0.00%	4.80%	0.00%	0.00%	0.00%
pp65:465-473	EEDTDEDSD	0.00%	0.00%	0.00%	5.76%	0.00%	0.00%	0.00%	0.00%	0.00%	0.00%
pp65:482-490	FTWPPWQAG	0.00%	3.99%	0.00%	0.00%	0.00%	0.00%	0.00%	0.00%	0.00%	0.00%
pp65:492-500	LARNLVPMV	1.45%	0.00%	0.00%	0.00%	0.00%	0.00%	0.00%	0.00%	0.00%	0.00%
pp65:495-503	NLVPMVATV	4.75%	72.77%	0.00%	12.93%	22.48%	87.00%	18.43%	58.67%	0.00%	28.58%
pp65:503-511	VQGQNLKYQ	0.00%	0.00%	22.36%	0.00%	0.00%	0.00%	0.00%	0.00%	0.00%	0.00%
pp65:511-519	QEFFWDAND	0.00%	0.00%	0.00%	0.00%	0.00%	0.00%	0.00%	0.00%	0.00%	7.80%
pp65:512-520	EFFWDANDI	0.00%	5.70%	0.00%	0.00%	0.00%	0.00%	0.00%	0.00%	0.06%	4.63%
pp65:513-521	FFWDANDIY	0.00%	4.97%	0.00%	0.00%	0.00%	0.00%	0.00%	0.00%	0.00%	8.26%
pp65:514-522	FWDANDIYR	0.00%	0.00%	0.00%	0.00%	0.00%	0.00%	0.00%	0.00%	0.00%	3.04%
pp65:521-529	YRIFAELEG	0.00%	0.00%	2.93%	0.00%	0.00%	0.00%	0.33%	0.00%	0.00%	0.00%
pp65:524-532	FAELEGVWQ	0.00%	2.77%	0.00%	0.00%	0.00%	0.00%	0.05%	0.00%	0.00%	0.00%
pp65:544-552	QDALPGPCI	0.00%	0.34%	0.00%	0.00%	2.34%	0.00%	0.00%	0.00%	0.00%	0.00%
**Cumulative Specific SFU (100%)**		**1185**	**424**	**2226**	**683**	**418**	**161**	**1450**	**1111**	**746**	**1103**
