# Supplementary material for: Discordance Between the Predicted Versus the Actually Recognized CD8+ T Cell Epitopes of HCMV pp65 Antigen and Aleatory Epitope Dominance
Source: Front Immunol. 2021 Feb 9;11:618428. doi: 10.3389/fimmu.2020.618428 (PMC7900545; doi:10.3389/fimmu.2020.618428)
Supplement: Supplementary file 6 [file Table_5.pdf]

Peptides Tested		Inidiviudal Subjets' CD8+ T Cell Response (SFU per 300,000 PBMC)									
Sequence	Peptide Name	ID 1	ID 2	ID 3	ID 4	ID 5	ID 6	ID 7	ID 8	ID 9	ID 10
pp65:011-019	MISVLGPIS	0	2	0	1	2	0	51	24	1	2
pp65:021-029	HVLKAVFSR	2	0	1	1	1	2	46	6	3	1
pp65:028-036	SRGDTPVLP	1	0	1	3	1	2	2	20	0	18
pp65:046-054	IHVRVSQPS	6	2	0	6	1	2	5	10	0	5
pp65:053-061	PSLILVSQY	1	1	5	31	1	0	52	8	1	3
pp65:066-074	TPCHRGDNQ	0	0	6	31	1	1	2	3	1	9
pp65:110-118	SIYVYALPL	8	0	5	9	0	0	1	18	3	13
pp65:117-125	PLKMLNIPS	7	1	5	7	1	2	2	2	1	5
pp65:120-128	MLNIPSINV	8	0	5	2	2	0	9	15	3	8
pp65:126-134	INVHHYPSA	0	0	5	8	1	5	6	5	3	18
pp65:134-142	AAERKHRHL	2	0	3	3	0	0	7	10	1	18
pp65:140-148	RHLPVADAV	6	0	2	9	2	1	2	11	5	7
pp65:162-170	TVSGLAWTR	7	1	7	9	2	2	2	5	8	3
pp65:174-182	QWKEPDVYY	10	0	15	2	1	0	2	5	5	2
pp65:193-201	VALRHVVCA	0	1	11	0	0	0	62	3	2	0
pp65:196-204	RHVVCAHEL	0	0	23	0	1	0	21	7	7	1
pp65:202-210	HELVCSMEN	0	0	1	0	8	2	9	10	2	7
pp65:209-217	ENTRATKMQ	1	0	5	0	7	5	2	3	5	3
pp65:210-218	NTRATKMQV	1	1	40	2	3	5	0	6	8	1
pp65:264-272	MRPHERNGF	3	0	2	6	8	0	9	3	1	2
pp65:277-285	PKNMIIKPG	2	0	9	32	0	0	1	10	2	0
pp65:285-293	GKISHIMLD	0	0	8	40	0	2	0	6	0	0
pp65:286-294	KISHIMLDV	1	2	3	16	8	1	0	3	5	13
pp65:289-297	HIMLDVAFT	0	2	3	26	1	0	3	10	3	0
pp65:290-298	IMLDVAFTS	2	9	2	14	5	1	3	6	5	9
pp65:293-302	DVAFTSHEH	1	0	3	8	0	1	6	5	2	18
pp65:301-309	HFGLLCPKS	2	0	1	18	0	0	7	1	0	20
pp65:304-312	LLCPKSIPG	1	0	6	8	1	5	3	8	2	21
pp65:306-314	CPKSIPGLS	1	1	5	15	1	1	10	7	1	18
pp65:311-319	PGLSISGNL	1	1	3	29	1	0	3	7	0	3
pp65;316-324	SGNLLMNGQ	0	6	8	2	1	2	2	9	3	29
pp65:332-340	AIRETVELR	6	0	3	7	1	0	5	5	7	17
pp65:335-343	ETVELRQYD	0	3	3	26	2	7	0	2	0	2
pp65:337-345	VELRQYDPV	0	0	14	31	0	0	2	7	11	3
pp65:340-348	RQYDPVAAL	6	7	5	6	0	2	1	2	5	21
pp65:341-349	QYDPVAALF	1	9	9	28	0	0	1	9	1	2
pp65:346-354	AALFFFDID	0	5	24	7	0	0	5	14	2	6
pp65:351-359	FDIDLLLQR	1	0	17	18	0	2	1	7	2	20
pp65:356-364	LLQRGPQYS	7	2	14	2	0	2	8	9	1	11
pp65:363-371	YSEHPTFTS	2	0	6	28	1	0	2	6	1	6
pp65:365-373	EHPTFTSQY	0	0	11	26	1	0	2	6	1	1
pp65:368-374	TFTSQYRIQ	10	1	26	8	0	7	9	14	13	15
pp65:380-388	EYRHTWDRH	1	2	9	28	0	1	9	3	5	9
pp65:383-391	HTWDRHDEG	0	0	25	13	1	0	16	3	2	2
pp65:393-401	AQGDDDVWT	6	0	1	1	0	2	9	5	1	2
pp65:412-420	TTERKTPRV	2	1	9	13	1	1	61	5	6	1
pp65:432-440	SAGRKRKSA	1	2	7	8	2	2	62	9	0	5
pp65:460-468	STVAPEEDT	2	8	6	29	9	0	30	20	10	6
pp65:466-474	EDTDEDSDN	1	0	5	29	0	7	2	5	3	10
pp65:472-480	SDNEIHNPA	0	0	5	6	1	23	3	8	5	13
pp65:477-485	HNPAVFTWP	0	8	5	3	1	1	0	7	5	5
pp65:486-494	PWQAGILAR	6	11	9	5	3	1	6	9	2	17
pp65:496-504	LVPMVATVQ	0	5	15	15	0	5	14	1	10	18
pp65:526-534	ELEGVWQPA	2	7	2	23	1	16	3	6	1	1
pp65:531-539	WQPAAQPKR	0	8	2	10	0	3	10	1	5	1
pp65:534-542	AAQPKRRRH	3	1	0	6	2	1	7	3	24	6
pp65:535-548	AQPKRRRHR	0	10	2	3	1	1	10	3	3	5
pp65:552-560	IASTPKKHR	1	9	2	18	0	1	14	6	14	3
Negative Controls and Cut Off Values For Response Categories	$\bar{x}$	1.0	0.8	4.2	3.9	3.9	1.8	6.5	8.4	5.4	3.2
	σ	1.0	1.3	3.6	4.4	0.5	2.4	5.6	5.6	3.7	2.7
	$\bar{x}*3\sigma$	3.9	4.6	14.9	17.1	5.5	8.8	23.3	25.2	16.6	11.3
	$\bar{x}*5\sigma$	5.8	7.2	22.1	25.9	6.5	13.5	34.4	36.3	24.0	16.8
	$\bar{x}*10\sigma$	10.7	13.7	40.0	47.8	9.1	25.3	62.4	64.2	42.5	30.3
	>100 SFU	>100	>100	>100	>100	>100	>100	>100	>100	>100	>100
