# Supplementary figures and images for: Discordance Between the Predicted Versus the Actually Recognized CD8+ T Cell Epitopes of HCMV pp65 Antigen and Aleatory Epitope Dominance
Source: Front Immunol. 2021 Feb 9;11:618428. doi: 10.3389/fimmu.2020.618428 (PMC7900545; doi:10.3389/fimmu.2020.618428)

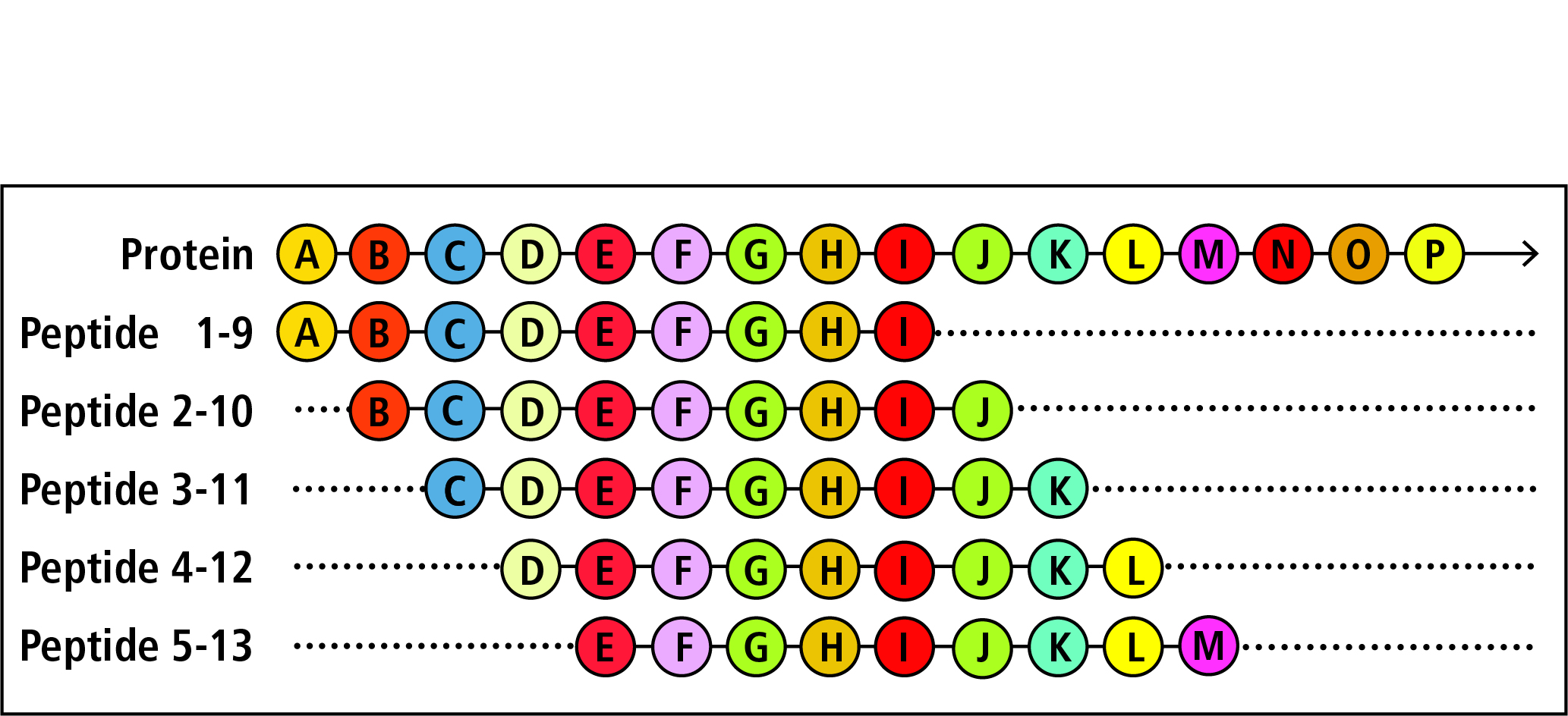

Supplement: Supplementary Figure 1 — Schematic representation of brute force CD8+ T cell epitope mapping. The amino acid sequence of the protein, illustrated on the top, is covered with nonamer peptides that walk the sequence in steps of single amino acids. [file Image_1.jpg]

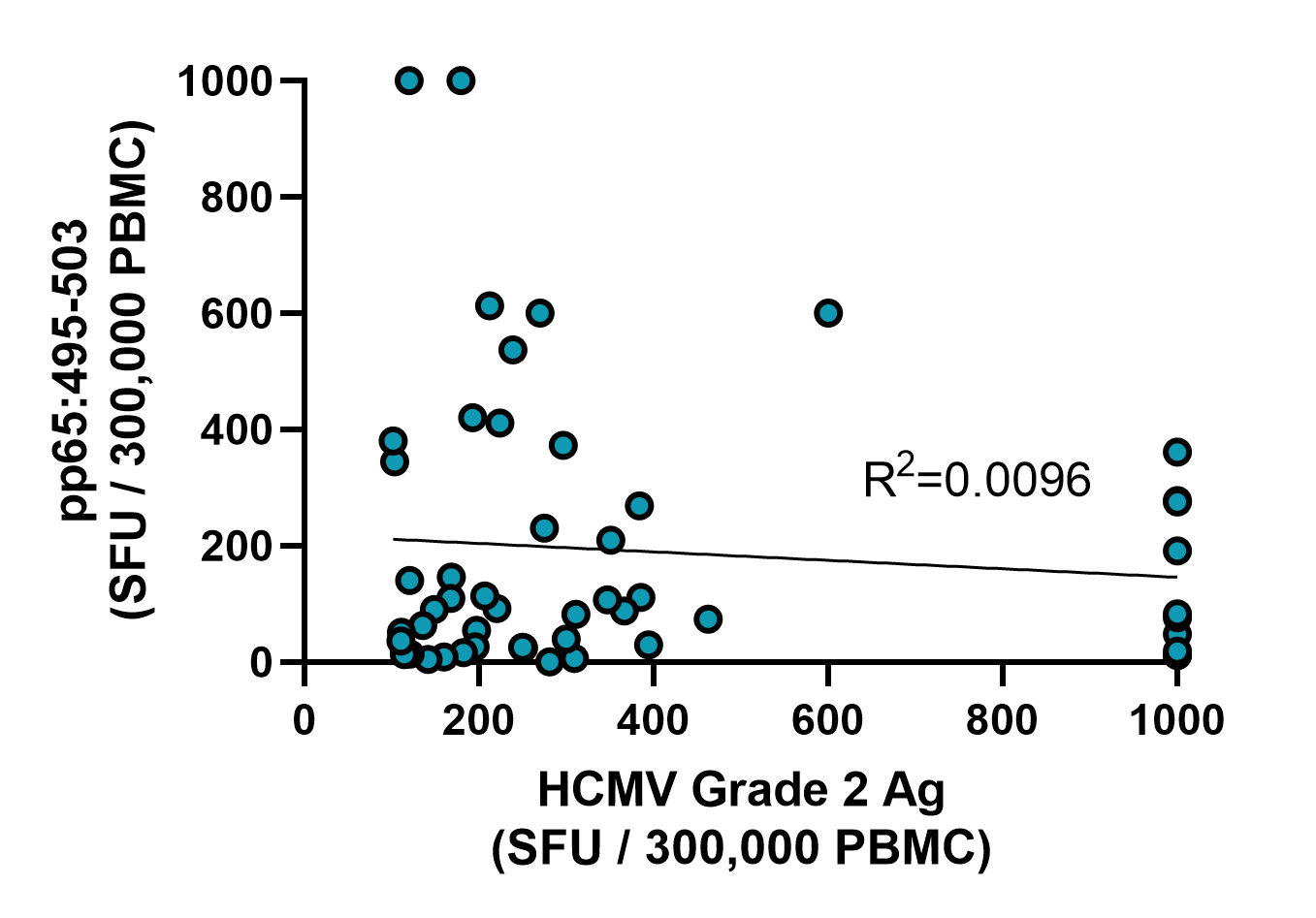

Supplement: Supplementary Figure 2 — Frequency of HCMV pp65495-503 peptide-specific CD8+ T cells vs. HCMV grade 2 antigen-reactive T cells. Fifty-two subjects were selected from the ePBMC database for being HLA-A*02:01 positive and responding to HCMV Grade 2 antigen with more than 100 SFU/300,000 PBMC. Each of these subjects’ PBMC (represented by a dot) were re-tested in an ImmunoSpot assay for the numbers of SFU triggered by HCMV Grade 2 antigen (shown on the X axis), and the numbers of SFU elicited by the pp65495-503 peptide (shown on the Y axis). No significant relationship was found by analysis through a simple linear regression. [file Image_2.tif]

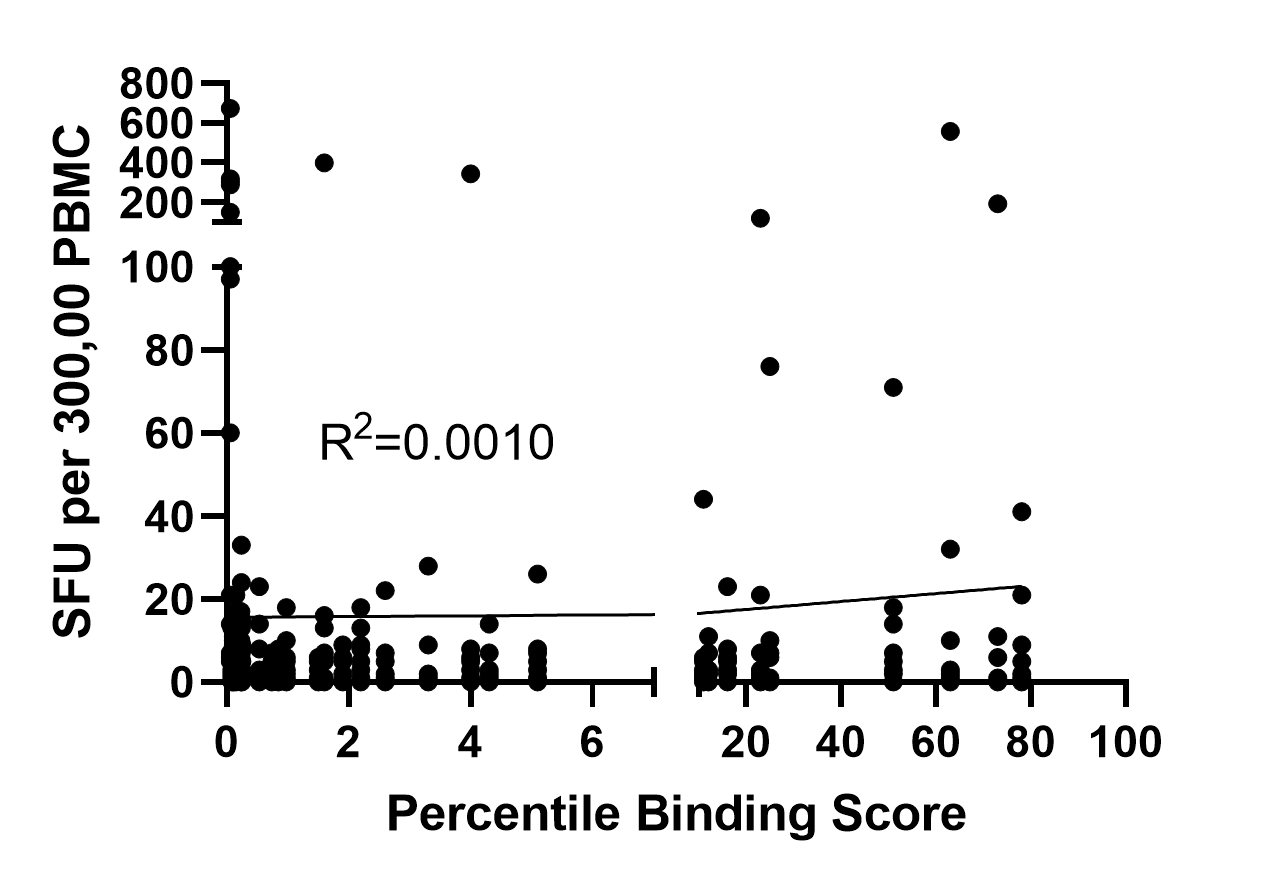

Supplement: Supplementary Figure 3 — HLA-A*02:01 binding ranking of previously defined HLA-A*02:01 restricted nonamer pp65 peptides vs. the SFU counts they induced in our cohort of HCMV positive, HLA-A*02:01 positive subjects. The numeric SFU data shown in S. Table 1 are plotted relative to their Percentile Binding Score as established run on the netMHCIpan search engine for predicting their binding to the HLA-A*02:01 allele. No significant relationship was found by analysis through a simple linear regression. [file Image_3.tif]

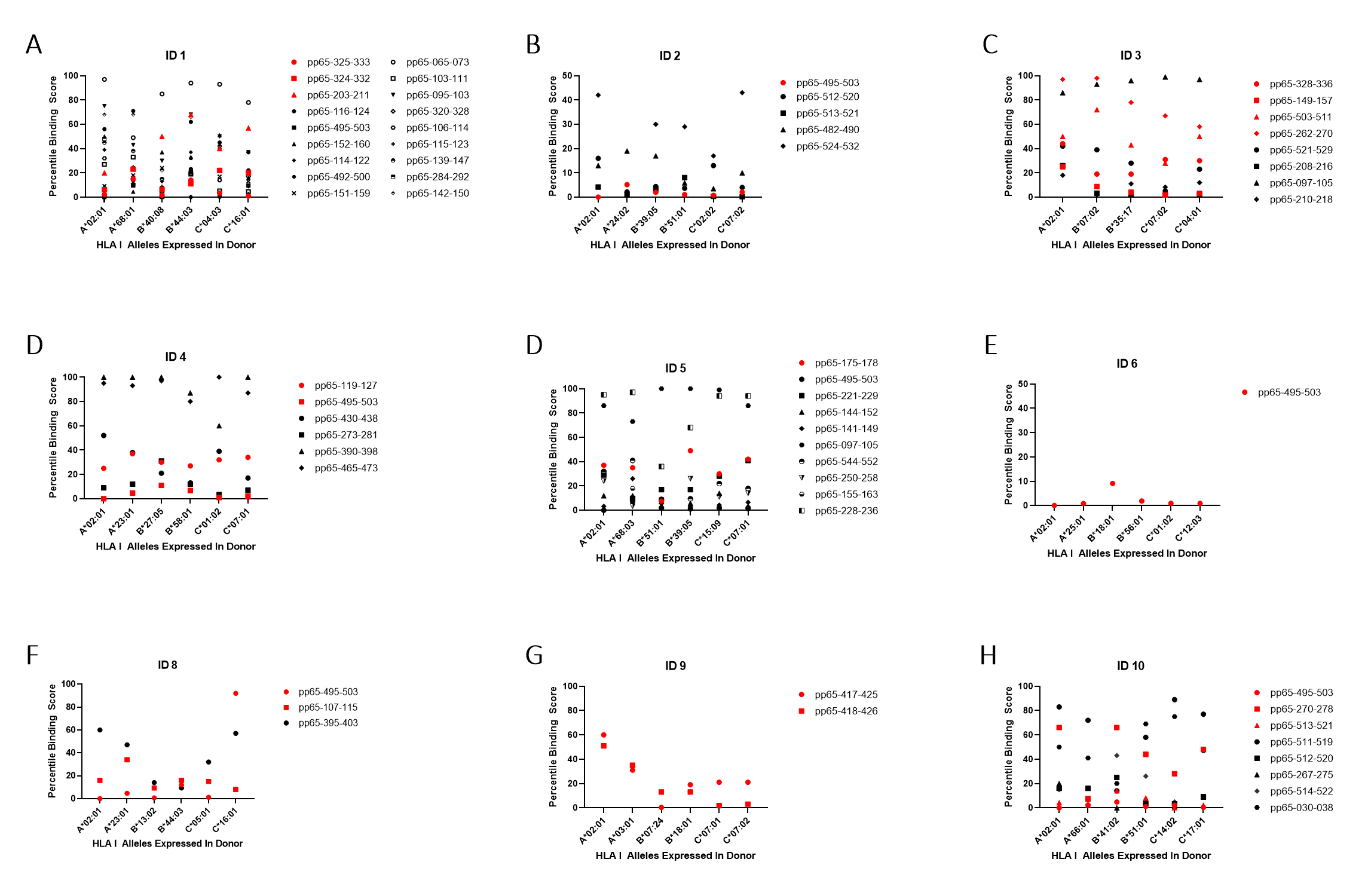

Supplement: Supplementary Figure 4 — Predicted vs. actual pp65 epitope recognition by CD8+ T cells. Data are shown for the subjects specified in each panel. For each subject his/her HLA class I alleles are specified (in the case of homozygosity the allele is listed once). Peptides that induced super-dominant responses in that individual are shown as red data points, dominant responses in black and weaker responses are not represented. The raw data for the peptide-induced SFU counts are listed in Table 1 . The IEBD Rank shown for each peptide and allele was established using the netMHCIpan search engine predicting the peptides’ binding score to the respective HLA allele, whereby a lower Percentile Binding Score binding score denotes better peptide binding. [file Image_4.tif]
